# Supplementary figures and images for: Arabidopsis eIF4E1 protects the translational machinery during TuMV infection and restricts virus accumulation
Source: PLoS Pathog. 2023 Nov 20;19(11):e1011417. doi: 10.1371/journal.ppat.1011417 (PMC10721207; doi:10.1371/journal.ppat.1011417)

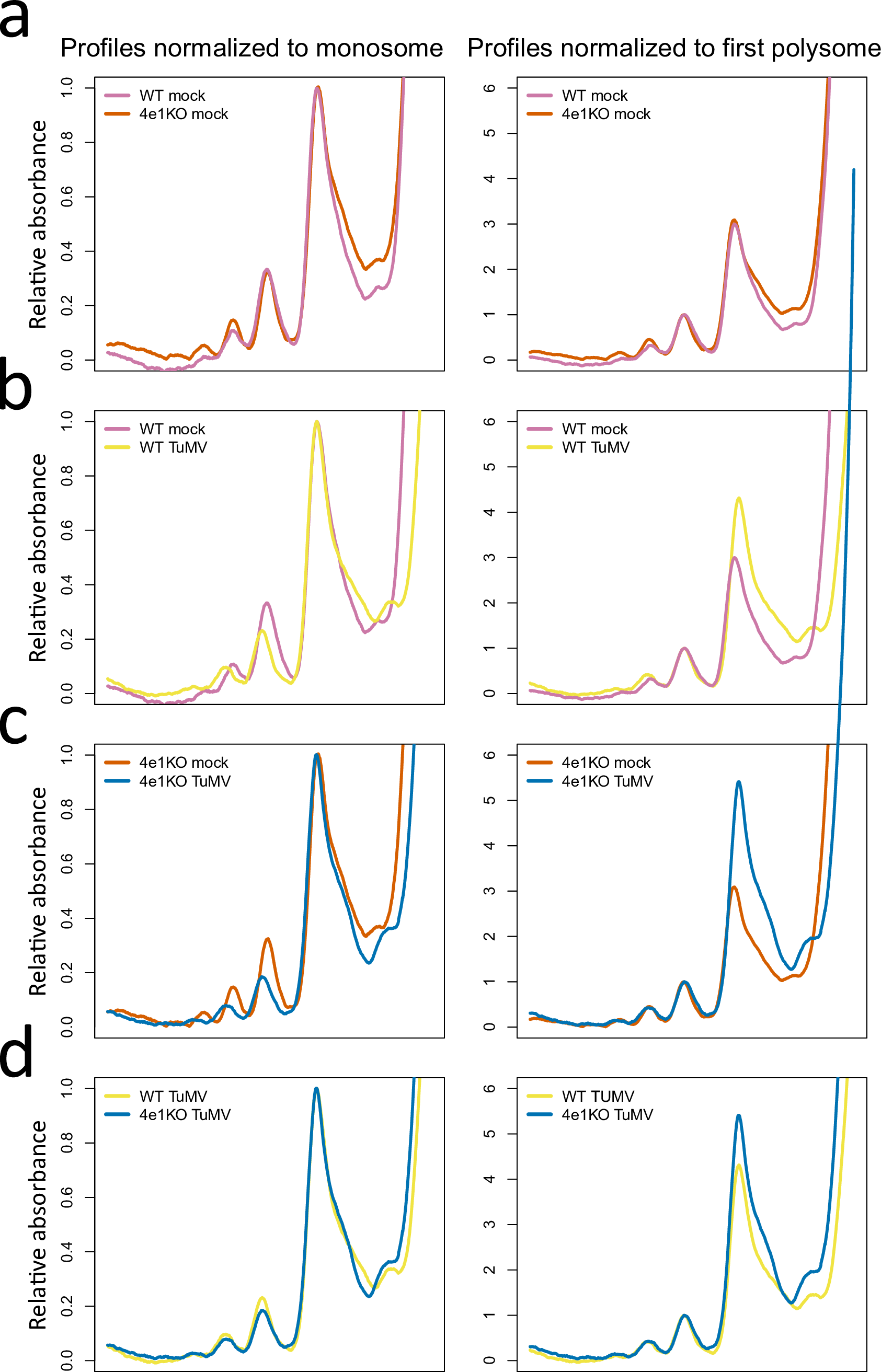

Supplement: S1 Fig — Comparison of monosome-normalized (left panels) and first polysome-normalized (right panels) polysome profiles for mock-inoculated wild-type and eif4e1KO plants (a), mock and TuMV-inoculated wild-type plants (b), mock and TuMV-inoculated eif4e1KO plants (c) and TuMV-inoculated wild-type and eif4e1KO plants 14 dpi. n = 3 biological replicates. WT = wild-type, 4e1KO = eif4e1KO. (TIF) [file ppat.1011417.s001.tif]

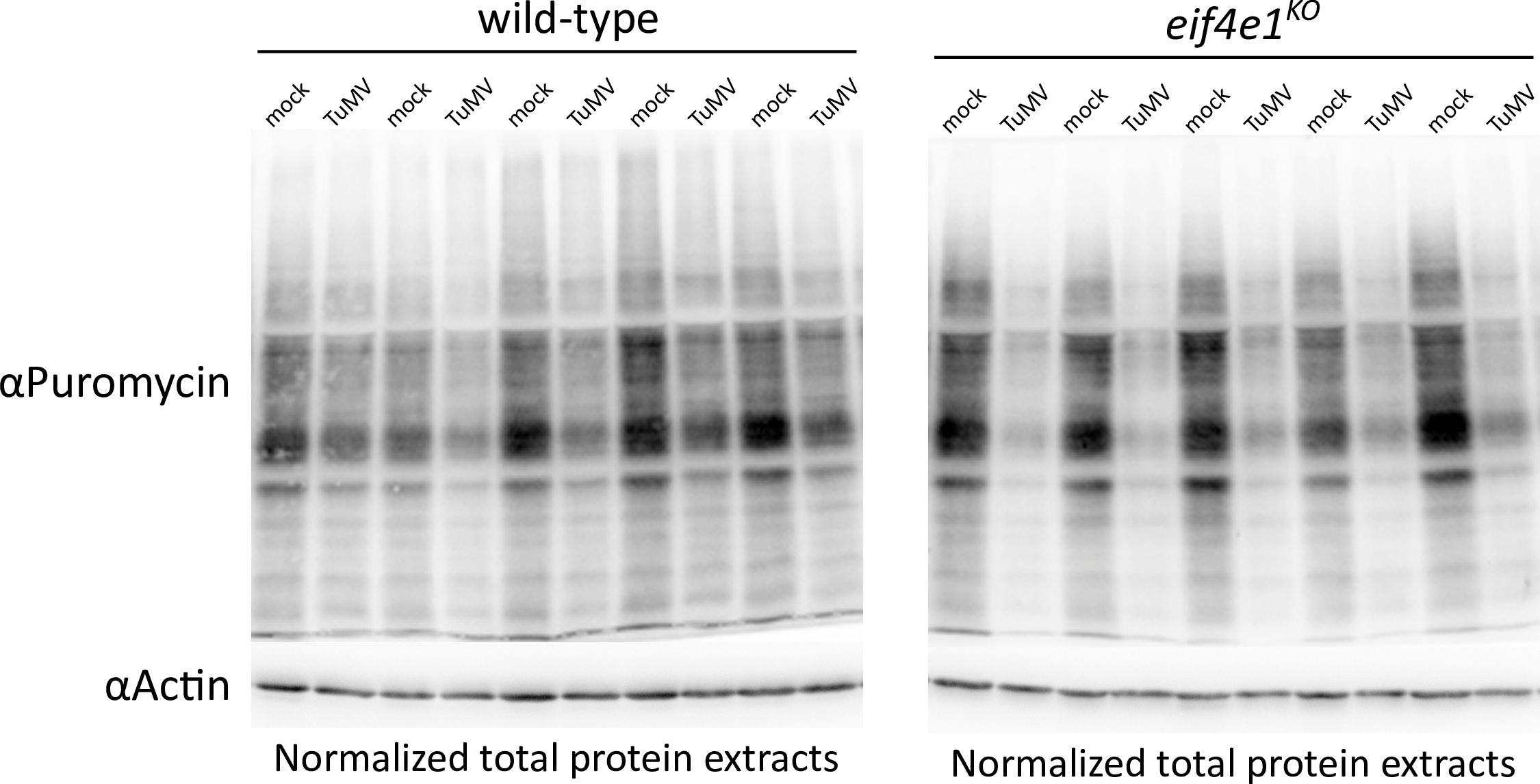

Supplement: S2 Fig — Western blot using anti-puromycin antibodies was performed on 20 ug of total protein extracts and equal protein levels were checked using anti-Actin antibodies. Each lane corresponds to an independent biological replicate. Membranes were imaged simultaneously. (TIF) [file ppat.1011417.s002.tif]

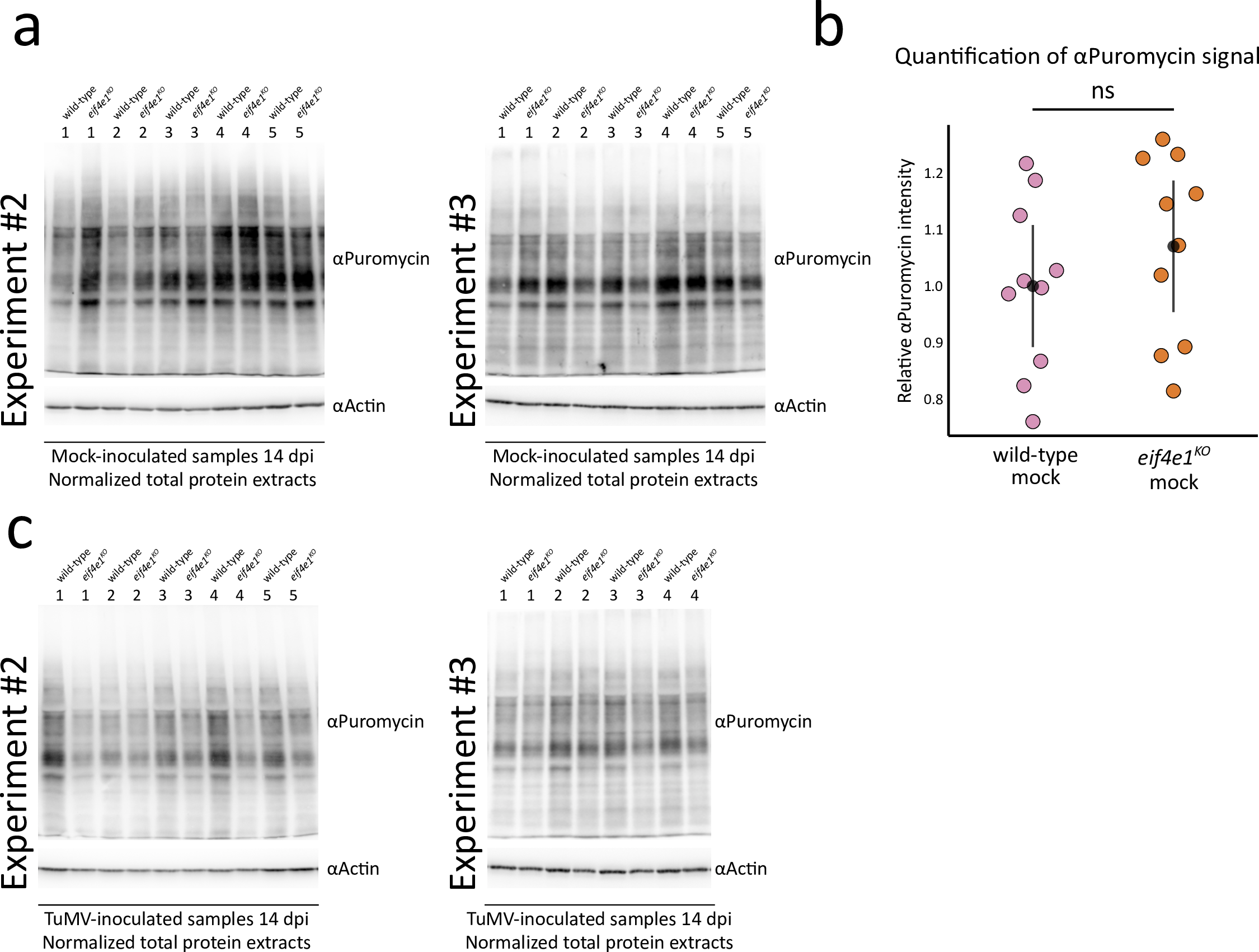

Supplement: S3 Fig — (a) Comparison of puromycin incorporation in mock-inoculated plants in 2 experimental repeats (b). Quantification of puromycin signal intensity from images shown in (a). Graphs show the 95% confidence intervals of the means obtained from 2 independent experimental repeats. n = 10 biological replicates. “ns” non-statistically significant difference (c) Comparison of puromycin incorporation in TuMV-inoculated plants in 3 experimental repeats. The photograph of the western blot associated with the third experimental repetition is displayed in Fig 1e. Western blot using anti-puromycin antibodies was performed on 20 ug of total protein extracts and equal protein levels were checked using anti-Actin antibodies. (TIF) [file ppat.1011417.s003.tif]

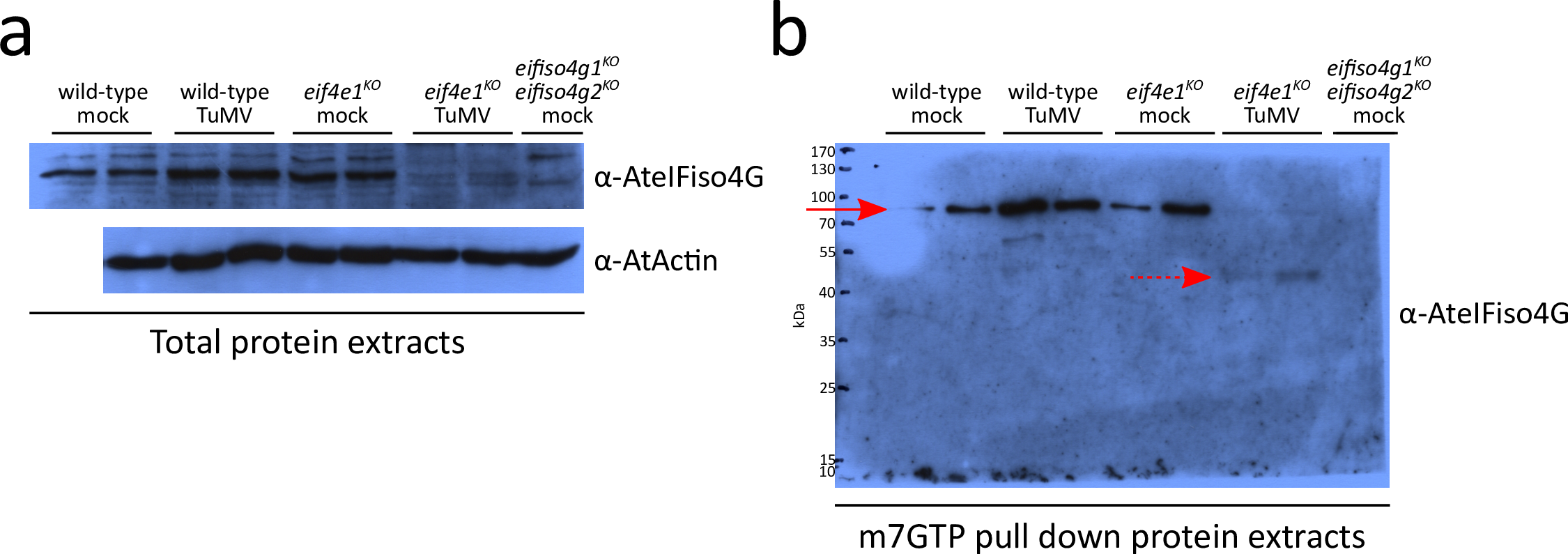

Supplement: S4 Fig — Western blot analyses of eIFiso4G1 accumulation in total protein extracts (a) or m7GTP pull down protein extracts (b) 14 dpi. Red arrowheads indicate the position of the full-length, 86 kDa, eIFiso4G1 protein. Dashed red arrowheads indicate the position of a 50 kDa protein product recognized by anti-eIFiso4G antibodies. Equal loading was checked on total protein extracts by western blot using anti-Actin antibodies. (TIF) [file ppat.1011417.s004.tif]

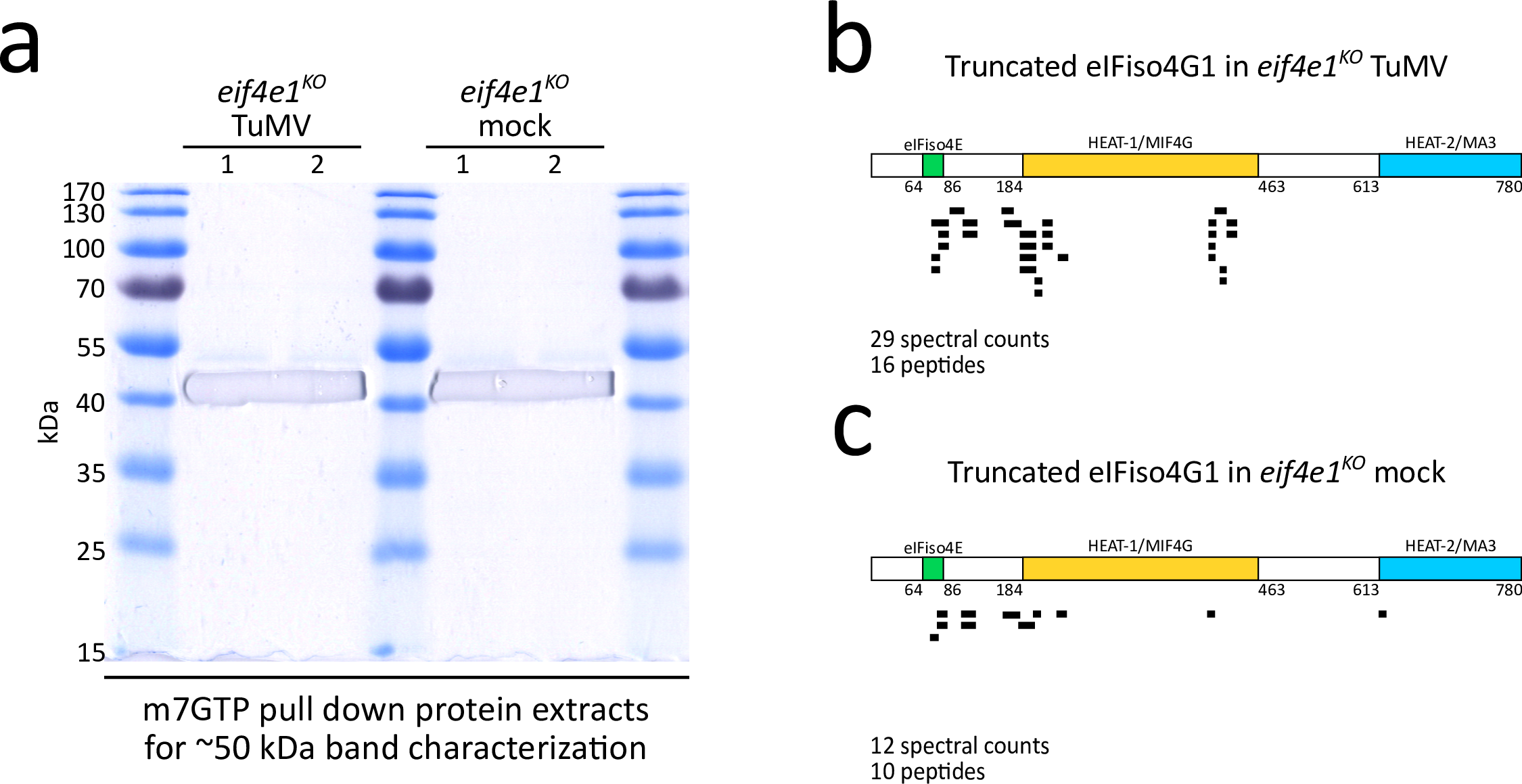

Supplement: S5 Fig — (a) Coomassie stained m7GTP-pull down protein extracts separated by SDS-PAGE electrophoresis. The protein content migrating between 40 and 55 kDa was excised from the gel and subjected to MS-based proteomic analyses for peptide identification. (b-c) Graphic representation of a truncated eIFiso4G1 protein product migrating at ~50 kDa in m7GTP pull down extracts obtained from eif4e1KO TuMV-inoculated plants (b) and mock-inoculated eif4e1KO plants (c) 14 dpi. Each horizontal bar corresponds to an identified peptide spectral count matching eIFiso4G1 sequence. The positions of the eIFiso4E-binding, HEAT1/MIF4G and HEAT2/MA3 domains, as well as total peptides and spectral counts identified in each experimental condition are given. n = 2 biological replicates. (TIF) [file ppat.1011417.s005.tif]

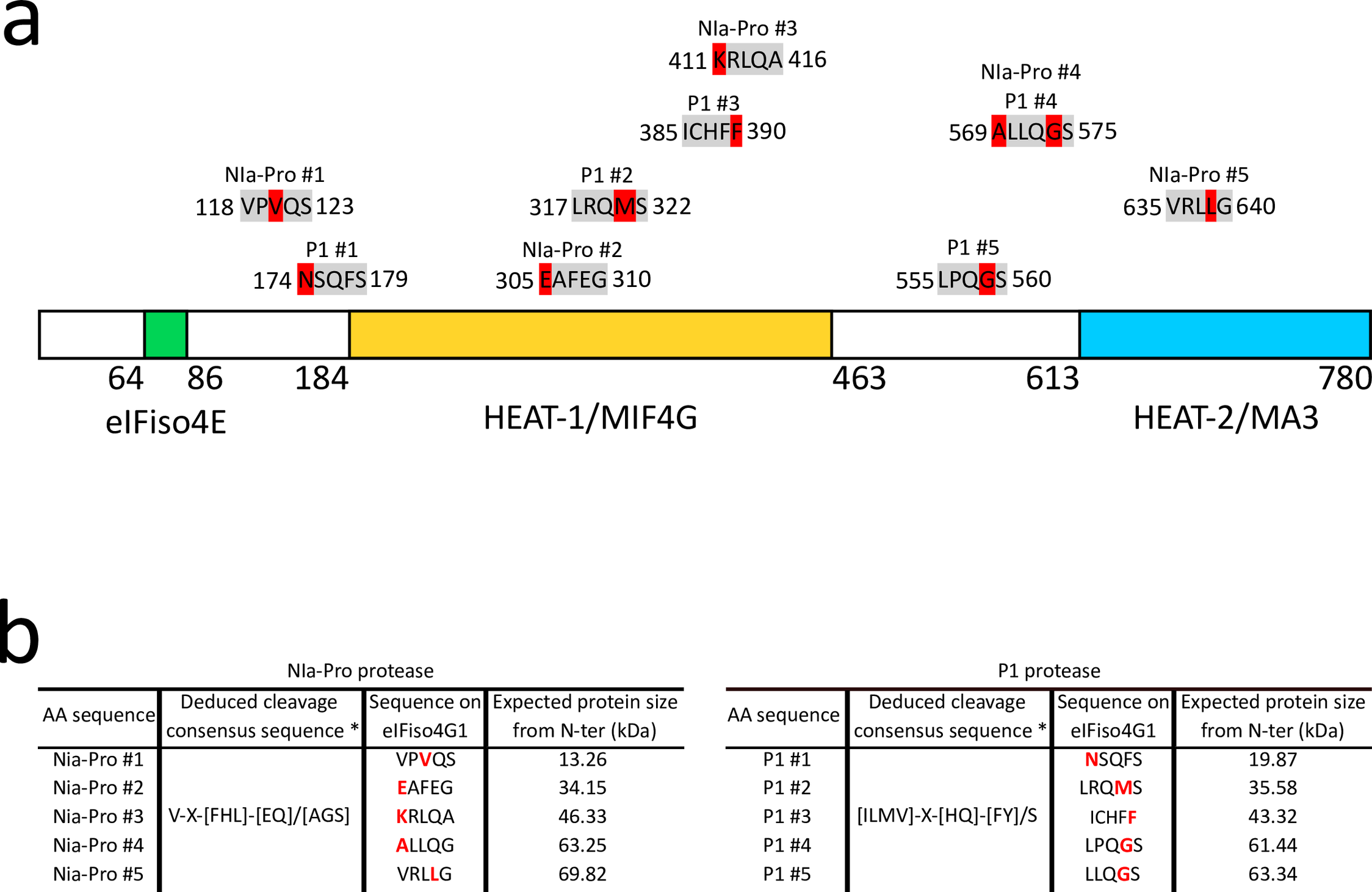

Supplement: S6 Fig — (a) A graphic representation of AteIFiso4G1 showing the positions of 10 amino acid sequences that differ by a single amino acid from the deduced consensus sequences recognized for cleavage by the Nia-Pro and P1 potyvirus proteases. For each of the amino acid sequences, the amino acid on eIFiso4G1 which differs from the consensus potyvirus cleavage sequence recognized is highlighted in red. No amino acid sequence on eIFiso4G1 bearing any similarity to the consensus cleavage sequence recognized by the potyvirus HC-Pro protease was found (b) A table summarizing the characteristics of the 10 amino acid sequences on eIFiso4G1 that differ by a single amino acid from the consensus potyvirus protease cleavage sequences. ‘X’ represents any amino acid, square brackets ([]) indicate that any of the amino acid inside is acceptable at the position, and slash (/) indicates the position of the scissile bond where the proteolytic cleavage occurs. The expected molecular weight of the potential cleavage products is shown for each of the amino acid sequences. The potyvirus proteases cleavage consensus sites were taken from (Goh and Hahn, 2021). The amino acid cleavage consensus sites on AteIFiso4G1 (Uniprot ID Q93ZT6) were searched using Fuzzpro https://www.bioinformatics.nl/cgi-bin/emboss/fuzzpro and the expected protein sizes were calculated using the Protein Molecular Weight tool https://www.bioinformatics.org/sms/prot_mw.html. (TIF) [file ppat.1011417.s006.tif]

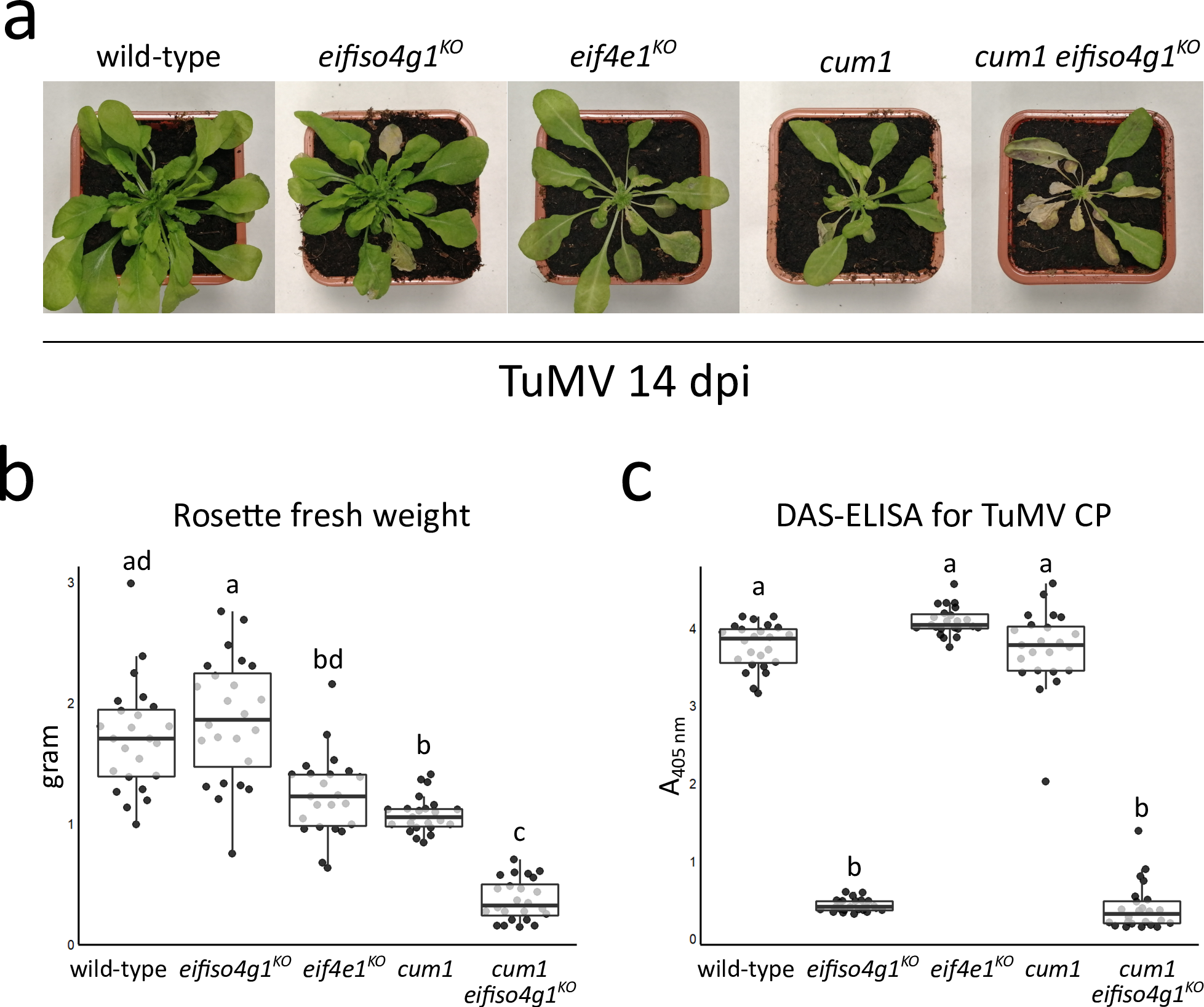

Supplement: S7 Fig — (a) Phenotypic comparison of TuMV-inoculated representative plants of each genotype 14 dpi. (b) Rosette fresh weight analysis of TuMV-inoculated plants 14 dpi. n = at least 23 biological replicates (c) Accumulation analysis of TuMV by DAS-ELISA 14 dpi. n = at least 23 biological replicates. Different letters depict significantly different groups identified by Kruskal–Wallis statistical tests at P < 0.05. (TIF) [file ppat.1011417.s007.tif]

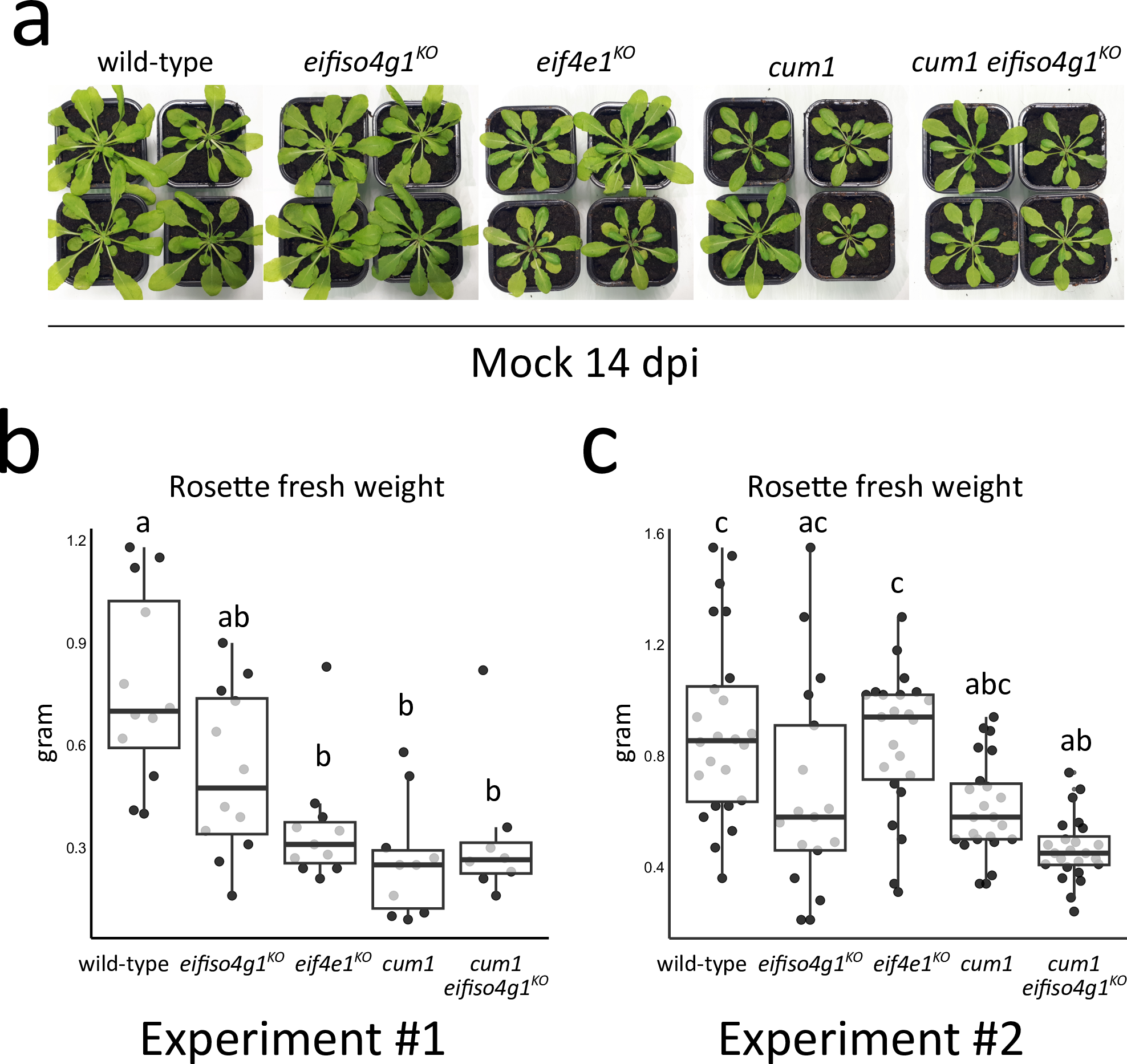

Supplement: S8 Fig — (a) Phenotypic comparison of mock-inoculated representative plants of each genotype 14 dpi. (b, c) Rosette fresh weight analysis of mock-inoculated plants 14 dpi in two independent experimental repeats. n = at least 8 biological replicates in (b) and at least 17 biological replicates in (c). Different letters depict significantly different groups identified by Kruskal–Wallis statistical tests at P < 0.05. (TIF) [file ppat.1011417.s008.tif]
