# Supplementary material for: Arabidopsis eIF4E1 protects the translational machinery during TuMV infection and restricts virus accumulation
Source: PLoS Pathog. 2023 Nov 20;19(11):e1011417. doi: 10.1371/journal.ppat.1011417 (PMC10721207; doi:10.1371/journal.ppat.1011417)
Supplement: S1 Supporting Dataset — (ZIP) [file ppat.1011417.s010.zip › Fig 1/Fig 1c-d (Polysome profiles and quantification data + script)/D-23-00790_Polysome_markdown_final.html]

Polysome profiling analysis


# Polysome profiling analysis

#### Delyan Zafirov and Cécile Lecampion

#### September, 2023

---

# 1 Introduction

The analysis presented here is a component of the data described in
the article “Arabidopsis eIF4E1 protects host translation and restricts
virus accumulation during TuMV infection”. The script conducts polysome
profiling analysis, including quantification of area under polysome and
monosome absorbance peaks, relative normalization of polysome and
monosome absorbance peaks, statistical analyses as well as graphical
representation of polysome absorbance profiles.

# 2 Data

The polysome profiling data is collected using a spectrophotometer
and saved in a ‘.csv’ file format. The file’s structure consists of
three columns, represented as follows:

0.0001041666692,0.1534467638,  
 0.000312499993,0.1535461843,  
0.0005208333605,0.1536204666,  
 …

For the analysis, only the values in the second column are retained -
this data will serve as the basis for the subsequent polysome profiling
analysis. The first two lines, as well as any extraneous lines at the
end of the file are omitted from the data processing. Additionally, any
text present in the file is excluded and only numeric values are
kept.

# 3 Data analysis

The data analysis is performed using a custom R function called
f\_load\_polysome\_csv. This function loads the ‘.csv’ file containing
polysome profiling data and returns a one-column dataframe that contains
only the second column - strings and empty lines are omitted. The column
is named using the parameter colName provided by the user.

```
#-------------------------------------------------------------------------------------
f_load_polysome_csv <- function(csvFile, colName) {
  lines <- readLines(csvFile)
  linesNbToImport <- grep("^\\d+\\.\\d+,\\d+\\.\\d+,", lines, perl = TRUE)
  df <- read.table(text = lines[linesNbToImport], header = FALSE, sep = ",", dec = ".")
  df <- as.data.frame(df$V2)
  colnames(df) <- c(colName)
  return(df)
}
```

## 3.1 Set variables for data analysis customization

In this section, we define the variables that allow customization of
the data analysis process.

```
# Define the working directory. The value of WORKING_DIRECTORY should be specified to your desired directory path.

WORKING_DIRECTORY <- "C:/Users/Delyan/Desktop/Manuscript iso4G submission/Markdown/final polysome data"

# Define the limits of the X-axis and Y-axis for the calculation of surface area and the graphical representation of the absorbance profiles:

# X-axis
XMIN <- 1000
XMAX <- 5000

# Y-axis
YMIN <- 0
YMAX <- 0.3

# Define the area to search for the monosome peak in the absorbance profiles:
ZMONO <- 3000

# Define the range of points to be considered when calculating the mean of the replicates:
MEAN_START <- 500
MEAN_END <- 5800
```

## 3.2 Loading and vislualization of raw absorbance profiles

In this section, we load the raw data of absorbance profiles for
different samples and visualize them graphically. This will provide an
initial understanding of the raw absorbance profiles.

```
setwd(WORKING_DIRECTORY)

# Load data:

## Hereafter, 'col' refers to wild-type, 'f4e' to eif4e1KO, 
## ='mock' = mock-inoculated 14 dpi, 'TuMV' = 'TuMV-inoculated 14 dpi'

col_mock1 <- f_load_polysome_csv("Col_mock1.csv", "col_mock1")
col_mock2 <- f_load_polysome_csv("Col_mock2.csv", "col_mock2")
col_mock3 <- f_load_polysome_csv("Col_mock3.csv", "col_mock3")
col_tumv1 <- f_load_polysome_csv("Col_TuMV1.csv", "col_tumv1")
col_tumv2 <- f_load_polysome_csv("Col_TuMV2.csv", "col_tumv2")
col_tumv3 <- f_load_polysome_csv("Col_TuMV3.csv", "col_tumv3")
f4e_mock1 <- f_load_polysome_csv("f4e_mock1.csv", "f4e_mock1")
f4e_mock2 <- f_load_polysome_csv("f4e_mock2.csv", "f4e_mock2")
f4e_mock3 <- f_load_polysome_csv("f4e_mock3.csv", "f4e_mock3")
f4e_tumv1 <- f_load_polysome_csv("f4e_TUMV1.csv", "f4e_tumv1")
f4e_tumv2 <- f_load_polysome_csv("f4e_TUMV2.csv", "f4e_tumv2")
f4e_tumv3 <- f_load_polysome_csv("f4e_TUMV3.csv", "f4e_tumv3")
```

```
# Visualize the raw data absorbance profiles

plot(x = 1 : nrow(col_mock1), y = col_mock1$col_mock1, 
     type = "l", xlim = c(XMIN, XMAX), ylim = c(YMIN, YMAX), frame.plot = FALSE, xlab = "", ylab = "")
points(x = 1 : nrow(col_mock2), y = col_mock2$col_mock2, 
       type = "l", xlim = c(XMIN, XMAX), ylim = c(YMIN, YMAX), col = "blue" )
points(x = 1 : nrow(col_mock3), y = col_mock3$col_mock3, 
       type = "l", xlim = c(XMIN, XMAX), ylim = c(YMIN, YMAX), col = "green" )
points(x = 1 : nrow(col_tumv1), y = col_tumv1$col_tumv1, 
       type = "l", xlim = c(XMIN, XMAX), ylim = c(YMIN, YMAX), col = "pink" )
points(x = 1 : nrow(col_tumv2), y = col_tumv2$col_tumv2, 
       type = "l", xlim = c(XMIN, XMAX), ylim = c(YMIN, YMAX), col = "orange" )
points(x = 1 : nrow(col_tumv3), y = col_tumv3$col_tumv3, 
       type = "l", xlim = c(XMIN, XMAX), ylim = c(YMIN, YMAX), col = "cyan" )
points(x = 1 : nrow(f4e_mock1), y = f4e_mock1$f4e_mock1, 
       type = "l", xlim = c(XMIN, XMAX), ylim = c(YMIN, YMAX), col = "red" )
points(x = 1 : nrow(f4e_mock2), y = f4e_mock2$f4e_mock2, 
       type = "l", xlim = c(XMIN, XMAX), ylim = c(YMIN, YMAX), col = "red1" )
points(x = 1 : nrow(f4e_mock3), y = f4e_mock3$f4e_mock3, 
       type = "l", xlim = c(XMIN, XMAX), ylim = c(YMIN, YMAX), col = "seagreen" )
points(x = 1 : nrow(f4e_tumv1), y = f4e_tumv1$f4e_tumv1, 
       type = "l", xlim = c(XMIN, XMAX), ylim = c(YMIN, YMAX), col = "sandybrown" )
points(x = 1 : nrow(f4e_tumv2), y = f4e_tumv2$f4e_tumv2, 
       type = "l", xlim = c(XMIN, XMAX), ylim = c(YMIN, YMAX), col = "red4" )
points(x = 1 : nrow(f4e_tumv3), y = f4e_tumv3$f4e_tumv3, 
       type = "l", xlim = c(XMIN, XMAX), ylim = c(YMIN, YMAX), col = "salmon" )
```

fig.1 : Raw polysomes data

## 3.3 Data preparation

To facilitate comparison between the absorbance profiles, we now
align the monosome peak and the baseline of each curve. This data
preparation step ensures that the curves are synchronized, making it
easier to observe and analyze the relative changes in polysome and
monosome distribution.

### 3.3.1 Determine position of the monosome pic

To identify the position of the monosome peak, we need to locate the
maximum absorbance value within the range defined by ZMONO and XMAX for
each curve. The ‘which’ function is used to find the index of the
maximum value for each curve:

```
# arr.ind = TRUE return the index of the maximum

max_col_mock1 <- which(col_mock1 == max(col_mock1[ZMONO:XMAX,], na.rm = TRUE), arr.ind = TRUE)[1]
max_col_mock2 <- which(col_mock2 == max(col_mock2[ZMONO:XMAX,], na.rm = TRUE), arr.ind = TRUE)[1]
max_col_mock3 <- which(col_mock3 == max(col_mock3[ZMONO:XMAX,], na.rm = TRUE), arr.ind = TRUE)[1]
max_f4e_mock1 <- which(f4e_mock1 == max(f4e_mock1[ZMONO:XMAX,], na.rm = TRUE), arr.ind = TRUE)[1]
max_f4e_mock2 <- which(f4e_mock2 == max(f4e_mock2[ZMONO:XMAX,], na.rm = TRUE), arr.ind = TRUE)[1]
max_f4e_mock3 <- which(f4e_mock3 == max(f4e_mock3[ZMONO:XMAX,], na.rm = TRUE), arr.ind = TRUE)[1]
max_col_tumv1 <- which(col_tumv1 == max(col_tumv1[ZMONO:XMAX,], na.rm = TRUE), arr.ind = TRUE)[1]
max_col_tumv2 <- which(col_tumv2 == max(col_tumv2[ZMONO:XMAX,], na.rm = TRUE), arr.ind = TRUE)[1]
max_col_tumv3 <- which(col_tumv3 == max(col_tumv3[ZMONO:XMAX,], na.rm = TRUE), arr.ind = TRUE)[1]
max_f4e_tumv1 <- which(f4e_tumv1 == max(f4e_tumv1[ZMONO:XMAX,], na.rm = TRUE), arr.ind = TRUE)[1]
max_f4e_tumv2 <- which(f4e_tumv2 == max(f4e_tumv2[ZMONO:XMAX,], na.rm = TRUE), arr.ind = TRUE)[1]
max_f4e_tumv3 <- which(f4e_tumv3 == max(f4e_tumv3[ZMONO:XMAX,], na.rm = TRUE), arr.ind = TRUE)[1]
```

Next, we determine the position of the monosome peak by finding the
minimum value among the maximum positions of all curves:

```
mono_pic <- min(c(max_col_mock1, max_col_mock2, max_col_mock3, max_f4e_mock1, max_f4e_mock2, max_f4e_mock3,
                  max_col_tumv1, max_col_tumv2, max_col_tumv3, max_f4e_tumv1, max_f4e_tumv2, max_f4e_tumv3))
```

The position of the monosome pic is : **4085**

### 3.3.2 Alignment of monosome pic

To align the monosome peak of all curves, we remove the leading lines
from the data frame for each sample. The number of lines removed is
calculated based on the difference between the maximum position of each
curve (max\_col\_ or max\_f4e\_) and the mono\_pic:

```
col_mock1 <- as.data.frame(col_mock1[-c(1 : (max_col_mock1 - mono_pic)), ])
col_mock2 <- as.data.frame(col_mock2[-c(1 : (max_col_mock2 - mono_pic)), ])
col_mock3 <- as.data.frame(col_mock3[-c(1 : (max_col_mock3 - mono_pic)), ])
col_tumv1 <- as.data.frame(col_tumv1[-c(1 : (max_col_tumv1 - mono_pic)), ])
col_tumv2 <- as.data.frame(col_tumv2[-c(1 : (max_col_tumv2 - mono_pic)), ])
col_tumv3 <- as.data.frame(col_tumv3[-c(1 : (max_col_tumv3 - mono_pic)), ])
f4e_mock1 <- as.data.frame(f4e_mock1[-c(1 : (max_f4e_mock1 - mono_pic)), ])
f4e_mock2 <- as.data.frame(f4e_mock2[-c(1 : (max_f4e_mock2 - mono_pic)), ])
f4e_mock3 <- as.data.frame(f4e_mock3[-c(1 : (max_f4e_mock3 - mono_pic)), ])
f4e_tumv1 <- as.data.frame(f4e_tumv1[-c(1 : (max_f4e_tumv1 - mono_pic)), ])
f4e_tumv2 <- as.data.frame(f4e_tumv2[-c(1 : (max_f4e_tumv2 - mono_pic)), ])
f4e_tumv3 <- as.data.frame(f4e_tumv3[-c(1 : (max_f4e_tumv3 - mono_pic)), ])

# The results are then visualized to ensure that all curves are aligned at the monosome peak:

plot(x = 1 : nrow(col_mock1), y = col_mock1$col_mock1, 
     type = "l", xlim = c(XMIN, XMAX), ylim = c(YMIN, YMAX), frame.plot = FALSE, xlab = "", ylab = "")
points(x = 1 : nrow(col_mock2), y = col_mock2$col_mock2, 
       type = "l", xlim = c(XMIN, XMAX), ylim = c(YMIN, YMAX), col = "blue" )
points(x = 1 : nrow(col_mock3), y = col_mock3$col_mock3, 
       type = "l", xlim = c(XMIN, XMAX), ylim = c(YMIN, YMAX), col = "green" )
points(x = 1 : nrow(col_tumv1), y = col_tumv1$col_tumv1, 
       type = "l", xlim = c(XMIN, XMAX), ylim = c(YMIN, YMAX), col = "pink" )
points(x = 1 : nrow(col_tumv2), y = col_tumv2$col_tumv2, 
       type = "l", xlim = c(XMIN, XMAX), ylim = c(YMIN, YMAX), col = "orange" )
points(x = 1 : nrow(col_tumv3), y = col_tumv3$col_tumv3, 
       type = "l", xlim = c(XMIN, XMAX), ylim = c(YMIN, YMAX), col = "cyan" )
points(x = 1 : nrow(f4e_mock1), y = f4e_mock1$f4e_mock1, 
       type = "l", xlim = c(XMIN, XMAX), ylim = c(YMIN, YMAX), col = "red" )
points(x = 1 : nrow(f4e_mock2), y = f4e_mock2$f4e_mock2, 
       type = "l", xlim = c(XMIN, XMAX), ylim = c(YMIN, YMAX), col = "red1" )
points(x = 1 : nrow(f4e_mock3), y = f4e_mock3$f4e_mock3, 
       type = "l", xlim = c(XMIN, XMAX), ylim = c(YMIN, YMAX), col = "seagreen" )
points(x = 1 : nrow(f4e_tumv1), y = f4e_tumv1$f4e_tumv1, 
       type = "l", xlim = c(XMIN, XMAX), ylim = c(YMIN, YMAX), col = "sandybrown" )
points(x = 1 : nrow(f4e_tumv2), y = f4e_tumv2$f4e_tumv2, 
       type = "l", xlim = c(XMIN, XMAX), ylim = c(YMIN, YMAX), col = "red4" )
points(x = 1 : nrow(f4e_tumv3), y = f4e_tumv3$f4e_tumv3, 
       type = "l", xlim = c(XMIN, XMAX), ylim = c(YMIN, YMAX), col = "salmon" )
```

fig.2 : Polysome profiles aligned on monosomes peak

### 3.3.3 Alignment of the baseline

We now align the baseline of each absorbance profile. This process
involves detecting the minimum absorbance value around the monosome peak
and adjusting all data points accordingly.

```
# Дetect the minimum on the y axis around the monosome for each curve

col_mock1 <- as.data.frame(col_mock1$col_mock1 - min(col_mock1$col_mock1[ZMONO:XMAX]))
col_mock2 <- as.data.frame(col_mock2$col_mock2 - min(col_mock2$col_mock2[ZMONO:XMAX]))
col_mock3 <- as.data.frame(col_mock3$col_mock3 - min(col_mock3$col_mock3[ZMONO:XMAX]))
col_tumv1 <- as.data.frame(col_tumv1$col_tumv1 - min(col_tumv1$col_tumv1[ZMONO:XMAX]))
col_tumv2 <- as.data.frame(col_tumv2$col_tumv2 - min(col_tumv2$col_tumv2[ZMONO:XMAX]))
col_tumv3 <- as.data.frame(col_tumv3$col_tumv3 - min(col_tumv3$col_tumv3[ZMONO:XMAX]))
f4e_mock1 <- as.data.frame(f4e_mock1$f4e_mock1 - min(f4e_mock1$f4e_mock1[ZMONO:XMAX]))
f4e_mock2 <- as.data.frame(f4e_mock2$f4e_mock2 - min(f4e_mock2$f4e_mock2[ZMONO:XMAX]))
f4e_mock3 <- as.data.frame(f4e_mock3$f4e_mock3 - min(f4e_mock3$f4e_mock3[ZMONO:XMAX]))
f4e_tumv1 <- as.data.frame(f4e_tumv1$f4e_tumv1 - min(f4e_tumv1$f4e_tumv1[ZMONO:XMAX]))
f4e_tumv2 <- as.data.frame(f4e_tumv2$f4e_tumv2 - min(f4e_tumv2$f4e_tumv2[ZMONO:XMAX]))
f4e_tumv3 <- as.data.frame(f4e_tumv3$f4e_tumv3 - min(f4e_tumv3$f4e_tumv3[ZMONO:XMAX]))

# Visualize the results

plot(x = 1 : nrow(col_mock1), y = col_mock1$col_mock1, 
     type = "l", xlim = c(XMIN, XMAX), ylim = c(YMIN, YMAX), frame.plot = FALSE, xlab = "", ylab = "")
points(x = 1 : nrow(col_mock2), y = col_mock2$col_mock2, 
       type = "l", xlim = c(XMIN, XMAX), ylim = c(YMIN, YMAX), col = "blue" )
points(x = 1 : nrow(col_mock3), y = col_mock3$col_mock3, 
       type = "l", xlim = c(XMIN, XMAX), ylim = c(YMIN, YMAX), col = "green" )
points(x = 1 : nrow(col_tumv1), y = col_tumv1$col_tumv1, 
       type = "l", xlim = c(XMIN, XMAX), ylim = c(YMIN, YMAX), col = "pink" )
points(x = 1 : nrow(col_tumv2), y = col_tumv2$col_tumv2, 
       type = "l", xlim = c(XMIN, XMAX), ylim = c(YMIN, YMAX), col = "orange" )
points(x = 1 : nrow(col_tumv3), y = col_tumv3$col_tumv3, 
       type = "l", xlim = c(XMIN, XMAX), ylim = c(YMIN, YMAX), col = "cyan" )
points(x = 1 : nrow(f4e_mock1), y = f4e_mock1$f4e_mock1, 
       type = "l", xlim = c(XMIN, XMAX), ylim = c(YMIN, YMAX), col = "red" )
points(x = 1 : nrow(f4e_mock2), y = f4e_mock2$f4e_mock2, 
       type = "l", xlim = c(XMIN, XMAX), ylim = c(YMIN, YMAX), col = "red1" )
points(x = 1 : nrow(f4e_mock3), y = f4e_mock3$f4e_mock3, 
       type = "l", xlim = c(XMIN, XMAX), ylim = c(YMIN, YMAX), col = "seagreen" )
points(x = 1 : nrow(f4e_tumv1), y = f4e_tumv1$f4e_tumv1, 
       type = "l", xlim = c(XMIN, XMAX), ylim = c(YMIN, YMAX), col = "sandybrown" )
points(x = 1 : nrow(f4e_tumv2), y = f4e_tumv2$f4e_tumv2, 
       type = "l", xlim = c(XMIN, XMAX), ylim = c(YMIN, YMAX), col = "red4" )
points(x = 1 : nrow(f4e_tumv3), y = f4e_tumv3$f4e_tumv3, 
       type = "l", xlim = c(XMIN, XMAX), ylim = c(YMIN, YMAX), col = "salmon" )
```

fig.3 : Polysome profiles aligned at baseline

# 4 Quantification of polysomes profiles

In this step, we quantify the absorbance profiles by keeping only the
significant points within the specified range defined by MEAN\_START and
MEAN\_END. These points will be used for further analysis.

```
# Keep only significant points within the specified range
sens_col_mock1 <- ((col_mock1$col_mock1[MEAN_START : MEAN_END]))
sens_col_mock2 <- ((col_mock2$col_mock2[MEAN_START : MEAN_END]))
sens_col_mock3 <- ((col_mock3$col_mock3[MEAN_START : MEAN_END]))
sens_col_tumv1 <- ((col_tumv1$col_tumv1[MEAN_START : MEAN_END]))
sens_col_tumv2 <- ((col_tumv2$col_tumv2[MEAN_START : MEAN_END]))
sens_col_tumv3 <- ((col_tumv3$col_tumv3[MEAN_START : MEAN_END]))
sens_f4e_mock1 <- ((f4e_mock1$f4e_mock1[MEAN_START : MEAN_END]))
sens_f4e_mock2 <- ((f4e_mock2$f4e_mock2[MEAN_START : MEAN_END]))
sens_f4e_mock3 <- ((f4e_mock3$f4e_mock3[MEAN_START : MEAN_END]))
sens_f4e_tumv1 <- ((f4e_tumv1$f4e_tumv1[MEAN_START : MEAN_END]))
sens_f4e_tumv2 <- ((f4e_tumv2$f4e_tumv2[MEAN_START : MEAN_END]))
sens_f4e_tumv3 <- ((f4e_tumv3$f4e_tumv3[MEAN_START : MEAN_END]))
```

## 4.1 Smoothing

Next, we apply the adjacent averaging method to smooth the
curves.

```
if (!requireNamespace("forecast", quietly = TRUE)) { install.packages("forecast") }
```

```
## Registered S3 method overwritten by 'quantmod':
##   method            from
##   as.zoo.data.frame zoo
```

```
library(forecast)
# Warning, function ma() convert data to time series, it is necessary to force numeric

col_mock1_smothed <- as.numeric(ma(sens_col_mock1, 
                                   order = 50, centre = TRUE))
col_mock2_smothed <- as.numeric(ma(sens_col_mock2, 
                                   order = 50, centre = TRUE))
col_mock3_smothed <- as.numeric(ma(sens_col_mock3, 
                                   order = 50, centre = TRUE))
col_tumv1_smothed <- as.numeric(ma(sens_col_tumv1, 
                                   order = 50, centre = TRUE))
col_tumv2_smothed <- as.numeric(ma(sens_col_tumv2, 
                                   order = 50, centre = TRUE))
col_tumv3_smothed <- as.numeric(ma(sens_col_tumv3, 
                                   order = 50, centre = TRUE))
f4e_mock1_smothed <- as.numeric(ma(sens_f4e_mock1, 
                                   order = 50, centre = TRUE))
f4e_mock2_smothed <- as.numeric(ma(sens_f4e_mock2, 
                                   order = 50, centre = TRUE))
f4e_mock3_smothed <- as.numeric(ma(sens_f4e_mock3, 
                                   order = 50, centre = TRUE))
f4e_tumv1_smothed <- as.numeric(ma(sens_f4e_tumv1, 
                                   order = 50, centre = TRUE))
f4e_tumv2_smothed <- as.numeric(ma(sens_f4e_tumv2, 
                                   order = 50, centre = TRUE))
f4e_tumv3_smothed <- as.numeric(ma(sens_f4e_tumv3, 
                                   order = 50, centre = TRUE))

# The smoothed data is combined into a new data frame named col_f4e_poly2_smoothed. The resulting data frame contains the smoothed absorbance profiles for all samples.

col_f4e_poly2_smoothed <- as.data.frame(cbind(col_mock1_smothed, col_mock2_smothed, col_mock3_smothed,
                                              col_tumv1_smothed, col_tumv2_smothed, col_tumv3_smothed,
                                              f4e_mock1_smothed, f4e_mock2_smothed, f4e_mock3_smothed,
                                              f4e_tumv1_smothed, f4e_tumv2_smothed, f4e_tumv3_smothed))

# Visualize the smoothed absorbance profiles 

plot(x = 1 : nrow(col_f4e_poly2_smoothed), y = col_f4e_poly2_smoothed$col_mock1_smothed,
     type = "l", xlim = c(XMIN, XMAX), ylim = c(YMIN, 0.1), col = "green", frame.plot = FALSE, 
     xlab = "", ylab = "")
points(x = 1 : nrow(col_f4e_poly2_smoothed), y = col_f4e_poly2_smoothed$col_mock2_smothed, 
       type = "l", xlim = c(XMIN, XMAX), ylim = c(YMIN, 0.1), col = "green1" )
points(x = 1 : nrow(col_f4e_poly2_smoothed), y = col_f4e_poly2_smoothed$col_mock3_smothed, 
       type = "l", xlim = c(XMIN, XMAX), ylim = c(YMIN, 0.1), col = "green2" )
points(x = 1 : nrow(col_f4e_poly2_smoothed), y = col_f4e_poly2_smoothed$col_tumv1_smothed, 
       type = "l", xlim = c(XMIN, XMAX), ylim = c(YMIN, 0.1), col = "olivedrab" )
points(x = 1 : nrow(col_f4e_poly2_smoothed), y = col_f4e_poly2_smoothed$col_tumv2_smothed, 
       type = "l", xlim = c(XMIN, XMAX), ylim = c(YMIN, 0.1), col = "olivedrab3" )
points(x = 1 : nrow(col_f4e_poly2_smoothed), y = col_f4e_poly2_smoothed$col_tumv3_smothed, 
       type = "l", xlim = c(XMIN, XMAX), ylim = c(YMIN, 0.1), col = "olivedrab4" )
points(x = 1 : nrow(col_f4e_poly2_smoothed), y = col_f4e_poly2_smoothed$f4e_mock1_smothed, 
       type = "l", xlim = c(XMIN, XMAX), ylim = c(YMIN, 0.1), col = "gold" )
points(x = 1 : nrow(col_f4e_poly2_smoothed), y = col_f4e_poly2_smoothed$f4e_mock2_smothed, 
       type = "l", xlim = c(XMIN, XMAX), ylim = c(YMIN, 0.1), col = "gold3" )
points(x = 1 : nrow(col_f4e_poly2_smoothed), y = col_f4e_poly2_smoothed$f4e_mock3_smothed, 
       type = "l", xlim = c(XMIN, XMAX), ylim = c(YMIN, 0.1), col = "gold4" )
points(x = 1 : nrow(col_f4e_poly2_smoothed), y = col_f4e_poly2_smoothed$f4e_tumv1_smothed, 
       type = "l", xlim = c(XMIN, XMAX), ylim = c(YMIN, 0.1), col = "red" )
points(x = 1 : nrow(col_f4e_poly2_smoothed), y = col_f4e_poly2_smoothed$f4e_tumv2_smothed, 
       type = "l", xlim = c(XMIN, XMAX), ylim = c(YMIN, 0.1), col = "red3" )
points(x = 1 : nrow(col_f4e_poly2_smoothed), y = col_f4e_poly2_smoothed$f4e_tumv3_smothed, 
       type = "l", xlim = c(XMIN, XMAX), ylim = c(YMIN, 0.1), col = "red4" )
```

fig.4 : Smoothed polysome profiles

## 4.2 Compute area under the curve

We now compute the area under the curve (AUC) for polysome and
monosome fractions in each absorbance profile. To do this, we delineate
the boundaries that distinguis the monosome and polysome fractons. The
determination of these boundaries is based on finding the minimum points
both before and after the monosome peak within each curve.

```
# Index of the minimum before the monosome peak

min1_col_mock1 <- which(col_f4e_poly2_smoothed == min(col_f4e_poly2_smoothed[ZMONO:(mono_pic - MEAN_START), 1], 
                                                      na.rm = TRUE),  arr.ind = TRUE)[1]
min1_col_mock2 <- which(col_f4e_poly2_smoothed == min(col_f4e_poly2_smoothed[ZMONO:(mono_pic - MEAN_START), 2], 
                                                      na.rm = TRUE),  arr.ind = TRUE)[1]
min1_col_mock3 <- which(col_f4e_poly2_smoothed == min(col_f4e_poly2_smoothed[ZMONO:(mono_pic - MEAN_START), 3], 
                                                      na.rm = TRUE),  arr.ind = TRUE)[1]
min1_col_tumv1 <- which(col_f4e_poly2_smoothed == min(col_f4e_poly2_smoothed[ZMONO:(mono_pic - MEAN_START), 4], 
                                                      na.rm = TRUE), arr.ind = TRUE)[1]
min1_col_tumv2 <- which(col_f4e_poly2_smoothed == min(col_f4e_poly2_smoothed[ZMONO:(mono_pic - MEAN_START), 5], 
                                                      na.rm = TRUE), arr.ind = TRUE)[1]
min1_col_tumv3 <- which(col_f4e_poly2_smoothed == min(col_f4e_poly2_smoothed[ZMONO:(mono_pic - MEAN_START), 6], 
                                                      na.rm = TRUE), arr.ind = TRUE)[1]
min1_f4e_mock1 <- which(col_f4e_poly2_smoothed == min(col_f4e_poly2_smoothed[ZMONO:(mono_pic - MEAN_START), 7], 
                                                      na.rm = TRUE), arr.ind = TRUE)[1]
min1_f4e_mock2 <- which(col_f4e_poly2_smoothed == min(col_f4e_poly2_smoothed[ZMONO:(mono_pic - MEAN_START), 8], 
                                                      na.rm = TRUE), arr.ind = TRUE)[1]
min1_f4e_mock3 <- which(col_f4e_poly2_smoothed == min(col_f4e_poly2_smoothed[ZMONO:(mono_pic - MEAN_START), 9], 
                                                      na.rm = TRUE), arr.ind = TRUE)[1]
min1_f4e_tumv1 <- which(col_f4e_poly2_smoothed == min(col_f4e_poly2_smoothed[ZMONO:(mono_pic - MEAN_START), 10], 
                                                      na.rm = TRUE), arr.ind = TRUE)[1]
min1_f4e_tumv2 <- which(col_f4e_poly2_smoothed == min(col_f4e_poly2_smoothed[ZMONO:(mono_pic - MEAN_START), 11], 
                                                      na.rm = TRUE), arr.ind = TRUE)[1]
min1_f4e_tumv3 <- which(col_f4e_poly2_smoothed == min(col_f4e_poly2_smoothed[ZMONO:(mono_pic - MEAN_START), 12], 
                                                      na.rm = TRUE), arr.ind = TRUE)[1]
# Index of the minimum after the monosome peak

min2_col_mock1 <- which(col_f4e_poly2_smoothed == min(col_f4e_poly2_smoothed[(mono_pic-MEAN_START): XMAX, 1], 
                                                      na.rm = TRUE),  arr.ind = TRUE)[1]
min2_col_mock2 <- which(col_f4e_poly2_smoothed == min(col_f4e_poly2_smoothed[(mono_pic-MEAN_START): XMAX, 2], 
                                                      na.rm = TRUE),  arr.ind = TRUE)[1]
min2_col_mock3 <- which(col_f4e_poly2_smoothed == min(col_f4e_poly2_smoothed[(mono_pic-MEAN_START): XMAX, 3], 
                                                      na.rm = TRUE),  arr.ind = TRUE)[1]
min2_col_tumv1 <- which(col_f4e_poly2_smoothed == min(col_f4e_poly2_smoothed[(mono_pic-MEAN_START): XMAX, 4], 
                                                      na.rm = TRUE), arr.ind = TRUE)[1]
min2_col_tumv2 <- which(col_f4e_poly2_smoothed == min(col_f4e_poly2_smoothed[(mono_pic-MEAN_START): XMAX, 5], 
                                                      na.rm = TRUE), arr.ind = TRUE)[1]
min2_col_tumv3 <- which(col_f4e_poly2_smoothed == min(col_f4e_poly2_smoothed[(mono_pic-MEAN_START): XMAX, 6], 
                                                      na.rm = TRUE), arr.ind = TRUE)[1]
min2_f4e_mock1 <- which(col_f4e_poly2_smoothed == min(col_f4e_poly2_smoothed[(mono_pic-MEAN_START): XMAX, 7], 
                                                      na.rm = TRUE), arr.ind = TRUE)[1]
min2_f4e_mock2 <- which(col_f4e_poly2_smoothed == min(col_f4e_poly2_smoothed[(mono_pic-MEAN_START): XMAX, 8], 
                                                      na.rm = TRUE), arr.ind = TRUE)[1]
min2_f4e_mock3 <- which(col_f4e_poly2_smoothed == min(col_f4e_poly2_smoothed[(mono_pic-MEAN_START): XMAX, 9], 
                                                      na.rm = TRUE), arr.ind = TRUE)[1]
min2_f4e_tumv1 <- which(col_f4e_poly2_smoothed == min(col_f4e_poly2_smoothed[(mono_pic-MEAN_START): XMAX, 10], 
                                                      na.rm = TRUE), arr.ind = TRUE)[1]
min2_f4e_tumv2 <- which(col_f4e_poly2_smoothed == min(col_f4e_poly2_smoothed[(mono_pic-MEAN_START): XMAX, 11], 
                                                      na.rm = TRUE), arr.ind = TRUE)[1]
min2_f4e_tumv3 <- which(col_f4e_poly2_smoothed == min(col_f4e_poly2_smoothed[(mono_pic-MEAN_START): XMAX, 12], 
                                                      na.rm = TRUE), arr.ind = TRUE)[1]


# Now we proceed to compute the area under the curve (AUC). To simplify the code, we include an index column in the data frame: 

col_f4e_poly2_smoothed$X <- c(1:nrow(col_f4e_poly2_smoothed))

# Load the package 'DescTools' 

if (!requireNamespace("DescTools", quietly = TRUE)) { install.packages("DescTools") }
library(DescTools)
```

```
## 
## Attaching package: 'DescTools'
```

```
## The following object is masked from 'package:forecast':
## 
##     BoxCox
```

```
col_mock1_AUC_poly <- AUC(x = col_f4e_poly2_smoothed[2000:min1_col_mock1, 13], 
                          y = col_f4e_poly2_smoothed[2000:min1_col_mock1, 1])
col_mock2_AUC_poly <- AUC(x = col_f4e_poly2_smoothed[2000:min1_col_mock2, 13], 
                          y = col_f4e_poly2_smoothed[2000:min1_col_mock2, 2])
col_mock3_AUC_poly <- AUC(x = col_f4e_poly2_smoothed[2000:min1_col_mock3, 13], 
                          y = col_f4e_poly2_smoothed[2000:min1_col_mock3, 3])
col_tumv1_AUC_poly <- AUC(x = col_f4e_poly2_smoothed[2000:min1_col_tumv1, 13], 
                          y = col_f4e_poly2_smoothed[2000:min1_col_tumv1, 4])
col_tumv2_AUC_poly <- AUC(x = col_f4e_poly2_smoothed[2000:min1_col_tumv2, 13], 
                          y = col_f4e_poly2_smoothed[2000:min1_col_tumv2, 5])
col_tumv3_AUC_poly <- AUC(x = col_f4e_poly2_smoothed[2000:min1_col_tumv3, 13], 
                          y = col_f4e_poly2_smoothed[2000:min1_col_tumv3, 6])
f4e_mock1_AUC_poly <- AUC(x = col_f4e_poly2_smoothed[2000:min1_f4e_mock1, 13], 
                          y = col_f4e_poly2_smoothed[2000:min1_f4e_mock1, 7])
f4e_mock2_AUC_poly <- AUC(x = col_f4e_poly2_smoothed[2000:min1_f4e_mock2, 13], 
                          y = col_f4e_poly2_smoothed[2000:min1_f4e_mock2, 8])
f4e_mock3_AUC_poly <- AUC(x = col_f4e_poly2_smoothed[2000:min1_f4e_mock3, 13], 
                          y = col_f4e_poly2_smoothed[2000:min1_f4e_mock3, 9])
f4e_tumv1_AUC_poly <- AUC(x = col_f4e_poly2_smoothed[2000:min1_f4e_tumv1, 13], 
                          y = col_f4e_poly2_smoothed[2000:min1_f4e_tumv1, 10])
f4e_tumv2_AUC_poly <- AUC(x = col_f4e_poly2_smoothed[2000:min1_f4e_tumv2, 13], 
                          y = col_f4e_poly2_smoothed[2000:min1_f4e_tumv2, 11])
f4e_tumv3_AUC_poly <- AUC(x = col_f4e_poly2_smoothed[2000:min1_f4e_tumv3, 13], 
                          y = col_f4e_poly2_smoothed[2000:min1_f4e_tumv3, 12])

col_mock1_AUC_mono <- AUC(x = col_f4e_poly2_smoothed[min1_col_mock1 : min2_col_mock1, 13], 
                          y = col_f4e_poly2_smoothed[min1_col_mock1 : min2_col_mock1, 1])
col_mock2_AUC_mono <- AUC(x = col_f4e_poly2_smoothed[min1_col_mock2 : min2_col_mock2, 13], 
                          y = col_f4e_poly2_smoothed[min1_col_mock2 : min2_col_mock2, 2])
col_mock3_AUC_mono <- AUC(x = col_f4e_poly2_smoothed[min1_col_mock3 : min2_col_mock3, 13], 
                          y = col_f4e_poly2_smoothed[min1_col_mock3 : min2_col_mock3, 3])
col_tumv1_AUC_mono <- AUC(x = col_f4e_poly2_smoothed[min1_col_tumv1 : min2_col_tumv1, 13], 
                          y = col_f4e_poly2_smoothed[min1_col_tumv1 : min2_col_tumv1, 4])
col_tumv2_AUC_mono <- AUC(x = col_f4e_poly2_smoothed[min1_col_tumv2 : min2_col_tumv2, 13], 
                          y = col_f4e_poly2_smoothed[min1_col_tumv2 : min2_col_tumv2, 5])
col_tumv3_AUC_mono <- AUC(x = col_f4e_poly2_smoothed[min1_col_tumv3 : min2_col_tumv3, 13], 
                          y = col_f4e_poly2_smoothed[min1_col_tumv3 : min2_col_tumv3, 6])
f4e_mock1_AUC_mono <- AUC(x = col_f4e_poly2_smoothed[min1_f4e_mock1 : min2_f4e_mock1, 13], 
                          y = col_f4e_poly2_smoothed[min1_f4e_mock1 : min2_f4e_mock1, 7])
f4e_mock2_AUC_mono <- AUC(x = col_f4e_poly2_smoothed[min1_f4e_mock2 : min2_f4e_mock2, 13], 
                          y = col_f4e_poly2_smoothed[min1_f4e_mock2 : min2_f4e_mock2, 8])
f4e_mock3_AUC_mono <- AUC(x = col_f4e_poly2_smoothed[min1_f4e_mock3 : min2_f4e_mock3, 13], 
                          y = col_f4e_poly2_smoothed[min1_f4e_mock3 : min2_f4e_mock3, 9])
f4e_tumv1_AUC_mono <- AUC(x = col_f4e_poly2_smoothed[min1_f4e_tumv1 : min2_f4e_tumv1, 13], 
                          y = col_f4e_poly2_smoothed[min1_f4e_tumv1 : min2_f4e_tumv1, 10])
f4e_tumv2_AUC_mono <- AUC(x = col_f4e_poly2_smoothed[min1_f4e_tumv2 : min2_f4e_tumv2, 13], 
                          y = col_f4e_poly2_smoothed[min1_f4e_tumv2 : min2_f4e_tumv2, 11])
f4e_tumv3_AUC_mono <- AUC(x = col_f4e_poly2_smoothed[min1_f4e_tumv3 : min2_f4e_tumv3, 13], 
                          y = col_f4e_poly2_smoothed[min1_f4e_tumv3 : min2_f4e_tumv3, 12])

# We create a new data frame "df2" to store the calculated AUC values for both the polysome and monosome fractions for each condition (Col_mock, Col_tumv, f4e_mock, and f4e_tumv).

cond <- rep(c("Col_mock", "Col_tumv", "f4e_mock", "f4e_tumv"), each = 3)
df2 <- as.data.frame(cond)
df2$poly <- c(col_mock1_AUC_poly, col_mock2_AUC_poly, col_mock3_AUC_poly, col_tumv1_AUC_poly, col_tumv2_AUC_poly, 
              col_tumv3_AUC_poly, f4e_mock1_AUC_poly, f4e_mock2_AUC_poly , f4e_mock3_AUC_poly, f4e_tumv1_AUC_poly, 
              f4e_tumv2_AUC_poly, f4e_tumv3_AUC_poly)
df2$mono <- c(col_mock1_AUC_mono, col_mock2_AUC_mono, col_mock3_AUC_mono, col_tumv1_AUC_mono, col_tumv2_AUC_mono, 
              col_tumv3_AUC_mono, f4e_mock1_AUC_mono, f4e_mock2_AUC_mono , f4e_mock3_AUC_mono, f4e_tumv1_AUC_mono, 
              f4e_tumv2_AUC_mono, f4e_tumv3_AUC_mono)


# We finally calculate the percentages of polysome (poly) and monosome (mono) fractions and store them in separate columns "poly_percent" and "mono_percent" in the "df2" data frame.

df2$poly_pourcent <- (df2$poly/(df2$poly+df2$mono))*100
df2$mono_pourcent <- (df2$mono/(df2$poly+df2$mono))*100

knitr::kable(df2,
             caption = "Area under the curve")
```

Area under the curve

| cond | poly | mono | poly\_pourcent | mono\_pourcent |
| --- | --- | --- | --- | --- |
| Col\_mock | 4.574039 | 19.48591 | 19.01101 | 80.98899 |
| Col\_mock | 5.538199 | 21.16857 | 20.73706 | 79.26294 |
| Col\_mock | 6.894643 | 21.02073 | 24.69838 | 75.30162 |
| Col\_tumv | 5.035755 | 30.44514 | 14.19287 | 85.80713 |
| Col\_tumv | 6.332263 | 27.60220 | 18.66027 | 81.33973 |
| Col\_tumv | 6.034759 | 28.17494 | 17.64049 | 82.35951 |
| f4e\_mock | 5.939920 | 24.50971 | 19.50736 | 80.49264 |
| f4e\_mock | 6.936836 | 24.13462 | 22.32543 | 77.67457 |
| f4e\_mock | 8.174863 | 26.62753 | 23.48937 | 76.51063 |
| f4e\_tumv | 5.628926 | 34.15399 | 14.14910 | 85.85090 |
| f4e\_tumv | 4.822012 | 31.55207 | 13.25672 | 86.74328 |
| f4e\_tumv | 6.938221 | 34.45898 | 16.76012 | 83.23988 |

# 5 Statistical analyses and graphic representation of polysome and monosome profiles quantification

We conduct statistical analyses and visualize the quantified polysome
surface areas using two approaches. In the first part, we represent the
quantifications as proportions of polysome or monosome surface area
relative to the total surface area for each experimental condition. In
the second part, we express the polysome profiles as a polysome to
monosome surface ratio relative to a control condition (here col\_mock,
i.e., mock-inoculated wild-type plants).

## 5.1 Proportion of polysomes and monosomes

Here express the quantifications as proportions, representing the
polysome or monosome surface area relative to the total surface area for
each experimental condition.

## 5.2 Statistical analysis

First, we check the normality of the data using the Shapiro-Wilk
normality test:

```
shapiro_df <- as.data.frame(c(shapiro.test(df2$poly_pourcent)$p.value, shapiro.test(df2$mono_pourcent)$p.value), row.names = c("Polysome", "Monosome")) %>% setNames(c("pvalue"))

knitr::kable(shapiro_df,
             caption = "Shapiro normality test")
```

Shapiro normality test

|  | pvalue |
| --- | --- |
| Polysome | 0.7670116 |
| Monosome | 0.7670116 |

If the p-value for the Shapiro-Wilk test is greater than 0.05, we can
conclude that the data follows a normal distribution.

**Data follow a normal distribution**

Next, we perform a two-sample t-test to compare the proportions of
polysomes between col and f4e conditions:

```
pval_col <- (t.test(df2$poly_pourcent[1:3], df2$poly_pourcent[4:6]))$p.value

pval_4e <- (t.test(df2$poly_pourcent[7:9], df2$poly_pourcent[10:12]))$p.value

student_df <- as.data.frame(c(pval_col, pval_4e), row.names = c("Col", "4E")) %>% setNames(c("pvalue"))
knitr::kable(student_df,
             caption = "Student test")
```

Student test

|  | pvalue |
| --- | --- |
| Col | 0.1007179 |
| 4E | 0.0115261 |

The p-values from the two-sample t-test allow us to assess if there
are significant differences in the proportions of polysomes between
conditions tested.

## 5.3 Visualization of results

To visualize the results of the analysis, we create a bar plot with
error bars to show the mean and standard deviation of polysome and
monosome proportions in each condition (Col\_mock, Col\_tumv, 4e\_mock,
4e\_tumv).

```
if (!requireNamespace("dplyr", quietly = TRUE)) { install.packages("dplyr") }
library(dplyr)
```

```
## 
## Attaching package: 'dplyr'
```

```
## The following objects are masked from 'package:stats':
## 
##     filter, lag
```

```
## The following objects are masked from 'package:base':
## 
##     intersect, setdiff, setequal, union
```

```
summary <- df2 %>%group_by(cond) %>% summarise(mean_poly = mean(poly_pourcent), sd_poly = sd(poly_pourcent), 
                                               mean_mono = mean(mono_pourcent), sd_mono = sd(mono_pourcent))
# convert data frame to long format
if (!requireNamespace("tidyr", quietly = TRUE)) { install.packages("tidyr") }
library(tidyr)
summary2 <- gather(summary, key = "fraction", value = "value", 2:5)

if (!requireNamespace("ggplot2", quietly = TRUE)) { install.packages("ggplot2") }
library(ggplot2)

ggplot(data=summary2[c(1:4,9:12),], aes(x=cond, y=value, fill=fraction)) +
  geom_bar(stat="identity") +
  geom_errorbar(aes(ymin=c(summary2$value[1:4]-summary2$value[5:8], 
                           (summary2$value[9:12]-summary2$value[13:16])+ summary2$value[1:4]) , 
                    ymax=c(summary2$value[1:4]+summary2$value[5:8], 
                           (summary2$value[9:12]+summary2$value[13:16])+ summary2$value[1:4]),
                    width=.2))+
  scale_y_continuous(breaks=c(0,25,50,75,100)) +
  scale_fill_manual(values=c("peachpuff", "thistle1"))+
  labs(title="",  x="", y = "%") +
  geom_curve(data = data.frame(x = 1, y = 106, 
                               xend = 2, yend = 106), mapping = aes(x = x,  y = y, xend = xend, yend = yend), 
             angle = 0L, curvature = 0L, 
             arrow = arrow(0L, unit(0.45, "inches"), "last", "closed"), 
             inherit.aes = FALSE) +
  geom_text(data = data.frame(x = 1.5, y = 109, 
                              label = paste("t.test, pval = ",round(pval_4e, 2) ) ), 
            mapping = aes(x = x, y = y, label = label), 
            inherit.aes = FALSE) +
  geom_curve(data = data.frame(x = 3, y = 106, 
                               xend = 4, yend = 106), mapping = aes(x = x,  y = y, xend = xend, yend = yend), 
             angle = 0L, curvature = 0L, 
             arrow = arrow(0L, unit(0.45, "inches"), "last", "closed"), 
             inherit.aes = FALSE) +
  geom_text(data = data.frame(x = 3.5, y = 109, 
                              label = paste("t.test, pval = ",round(pval_col, 2) ) ), 
            mapping = aes(x = x, y = y, label = label), 
            inherit.aes = FALSE) +
  theme_classic()
```

fig.5: Proportion of polysomes and monosomes

## 5.4 Polysome/monosome ratio relative to control

We represent the polysome profiles as a polysome-to-monosome surface
ratio relative to wild-type mock-inoculated plants (col\_mock).

```
df2$ratio <- (df2$poly/df2$mono)/mean(df2$poly[1:3]/df2$mono[1:3])

summary_ratio <- df2 %>%group_by(cond) %>% summarise(mean_ratio = mean(ratio), sd_ratio = sd(ratio))
```

### 5.4.1 Statistical analysis

First, we check the normality of the data using the Shapiro-Wilk
normality test:

```
shapiro_df_ratio <- as.data.frame(c(shapiro.test(df2$ratio)$p.value)) %>% setNames(c("pvalue"))

knitr::kable(shapiro_df,
             caption = "Shapiro normality test")
```

Shapiro normality test

|  | pvalue |
| --- | --- |
| Polysome | 0.7670116 |
| Monosome | 0.7670116 |

If the p-value for the Shapiro-Wilk test is greater than 0.05, we can
conclude that the data follows a normal distribution.

**Data follow a normal distribution**

Next, we conduct a Tukey test to determine significant differences in
the relative polysome/monosome ratio between the tested conditions:

```
if (!requireNamespace("rstatix", quietly = TRUE)) { install.packages("rstatix") }
library(rstatix)
```

```
## 
## Attaching package: 'rstatix'
```

```
## The following object is masked from 'package:stats':
## 
##     filter
```

```
res_tukey <- df2 %>% tukey_hsd(ratio~cond)
knitr::kable(res_tukey,
             caption = "Tukey test")
```

Tukey test


| term | group1 | group2 | null.value | estimate | conf.low | conf.high | p.adj | p.adj.signif |
| --- | --- | --- | --- | --- | --- | --- | --- | --- |
| cond | Col\_mock | Col\_tumv | 0 | -0.2612322 | -0.6030068 | 0.0805424 | 0.1450 | ns |
| cond | Col\_mock | f4e\_mock | 0 | 0.0150763 | -0.3266983 | 0.3568509 | 0.9990 | ns |
| cond | Col\_mock | f4e\_tumv | 0 | -0.3704333 | -0.7122079 | -0.0286587 | 0.0343 | \* |
| cond | Col\_tumv | f4e\_mock | 0 | 0.2763085 | -0.0654661 | 0.6180831 | 0.1190 | ns |
| cond | Col\_tumv | f4e\_tumv | 0 | -0.1092011 | -0.4509757 | 0.2325735 | 0.7410 | ns |
| cond | f4e\_mock | f4e\_tumv | 0 | -0.3855096 | -0.7272842 | -0.0437350 | 0.0282 | \* |

The p-values obtained from the Tukey test help us identify any
statistically significant variations in polysome/monosome ratio among
the experimental conditions.

### 5.4.2 Results visualization

The results are depicted graphically below:

```
ggplot(data=summary_ratio, aes(x=cond, y=mean_ratio)) +
  geom_bar(stat="identity", color = "lightblue", fill = "lightblue") +
  geom_errorbar(aes(ymin=summary_ratio$mean_ratio-summary_ratio$sd_ratio, 
                    ymax=summary_ratio$mean_ratio+summary_ratio$sd_ratio, 
                    width=.2))+
  geom_curve(data = data.frame(x = 1, y = 1.4, 
                               xend = 2, yend = 1.4), mapping = aes(x = x,  y = y, xend = xend, yend = yend), 
             angle = 0L, curvature = 0L, 
             arrow = arrow(0L, unit(0.45, "inches"), "last", "closed"), 
             inherit.aes = FALSE) +
  geom_text(data = data.frame(x = 1.5, y = 1.44, 
                              label = paste(res_tukey[1,9] ) ), 
            mapping = aes(x = x, y = y, label = label), 
            inherit.aes = FALSE)+
  geom_curve(data = data.frame(x = 1, y = 1.5, 
                               xend = 3, yend = 1.5), mapping = aes(x = x,  y = y, xend = xend, yend = yend), 
             angle = 0L, curvature = 0L, 
             arrow = arrow(0L, unit(0.45, "inches"), "last", "closed"), 
             inherit.aes = FALSE) +
  geom_text(data = data.frame(x = 2, y = 1.55, 
                              label = paste(res_tukey[2,9] ) ), 
            mapping = aes(x = x, y = y, label = label), 
            inherit.aes = FALSE)+
  geom_curve(data = data.frame(x = 1, y = 1.6, 
                               xend = 4, yend = 1.6), mapping = aes(x = x,  y = y, xend = xend, yend = yend), 
             angle = 0L, curvature = 0L, 
             arrow = arrow(0L, unit(0.45, "inches"), "last", "closed"), 
             inherit.aes = FALSE) +
  geom_text(data = data.frame(x = 2.5, y = 1.66, 
                              label = paste(res_tukey[3,9] ) ), 
            mapping = aes(x = x, y = y, label = label), 
            inherit.aes = FALSE)+
  geom_curve(data = data.frame(x = 2, y = 1.23, 
                               xend = 3, yend = 1.23), mapping = aes(x = x,  y = y, xend = xend, yend = yend), 
             angle = 0L, curvature = 0L, 
             arrow = arrow(0L, unit(0.45, "inches"), "last", "closed"), 
             inherit.aes = FALSE) +
  geom_text(data = data.frame(x = 2.5, y = 1.26, 
                              label = paste(res_tukey[4,9] ) ), 
            mapping = aes(x = x, y = y, label = label), 
            inherit.aes = FALSE)+
  geom_curve(data = data.frame(x = 2, y = 1.32, 
                               xend = 4, yend = 1.32), mapping = aes(x = x,  y = y, xend = xend, yend = yend), 
             angle = 0L, curvature = 0L, 
             arrow = arrow(0L, unit(0.45, "inches"), "last", "closed"), 
             inherit.aes = FALSE) +
  geom_text(data = data.frame(x = 3, y = 1.35, 
                              label = paste(res_tukey[5,9] ) ), 
            mapping = aes(x = x, y = y, label = label), 
            inherit.aes = FALSE)+
  geom_curve(data = data.frame(x = 3, y = 1.2, 
                               xend = 4, yend = 1.2), mapping = aes(x = x,  y = y, xend = xend, yend = yend), 
             angle = 0L, curvature = 0L, 
             arrow = arrow(0L, unit(0.45, "inches"), "last", "closed"), 
             inherit.aes = FALSE) +
  geom_text(data = data.frame(x = 3.5, y = 1.23, 
                              label = paste(res_tukey[6,9] ) ), 
            mapping = aes(x = x, y = y, label = label), 
            inherit.aes = FALSE)+
  theme_classic()
```

fig.6 : Polysome/monosome ratio relative to Col\_mock

# 6 Visualization of absorbance profiles

Hereafter, we provide the script for visualizing the obtained
polysome profiles. We first calculate the mean of the raw data obtained
from different replicates and organize the results in a data frame. We
then plot the absorbance profiles for each condition.

```
# Mean Calculation and plotting

mean_col_mock <- rowMeans(cbind(col_mock1$col_mock1[MEAN_START : MEAN_END], 
                                col_mock2$col_mock2[MEAN_START : MEAN_END],
                                col_mock3$col_mock3[MEAN_START : MEAN_END]))
mean_col_tumv <- rowMeans(cbind(col_tumv1$col_tumv1[MEAN_START : MEAN_END], 
                                col_tumv2$col_tumv2[MEAN_START : MEAN_END],
                                col_tumv3$col_tumv3[MEAN_START : MEAN_END]))
mean_f4e_mock <- rowMeans(cbind(f4e_mock1$f4e_mock1[MEAN_START : MEAN_END], 
                                f4e_mock2$f4e_mock2[MEAN_START : MEAN_END],
                                f4e_mock3$f4e_mock3[MEAN_START : MEAN_END]))
mean_f4e_tumv <- rowMeans(cbind(f4e_tumv1$f4e_tumv1[MEAN_START : MEAN_END], 
                                f4e_tumv2$f4e_tumv2[MEAN_START : MEAN_END],
                                f4e_tumv3$f4e_tumv3[MEAN_START : MEAN_END]))

col_f4e_poly2_mean <- as.data.frame(cbind(mean_col_mock, mean_f4e_mock, mean_col_tumv, mean_f4e_tumv))

plot(x = 1 : nrow(col_f4e_poly2_mean), y = col_f4e_poly2_mean$mean_col_mock,
     type = "l", xlim = c(XMIN, XMAX), ylim = c(YMIN, 0.1), col = "seagreen1", frame.plot = FALSE,
     xlab = "", ylab = "")
points(x = 1 : nrow(col_f4e_poly2_mean), y = col_f4e_poly2_mean$mean_col_tumv, 
       type = "l", xlim = c(XMIN, XMAX), ylim = c(YMIN, 0.1), col = "darkgreen" )
points(x = 1 : nrow(col_f4e_poly2_mean), y = col_f4e_poly2_mean$mean_f4e_mock, 
       type = "l", xlim = c(XMIN, XMAX), ylim = c(YMIN, 0.1), col = "goldenrod" )
points(x = 1 : nrow(col_f4e_poly2_mean), y = col_f4e_poly2_mean$mean_f4e_tumv, 
       type = "l", xlim = c(XMIN, XMAX), ylim = c(YMIN, 0.1), col = "red" )
```

fig.7 : Polysome profiles mean of replicates

Next, we apply the adjacent averaging method to smoothen the
data.

## 6.1 Smoothing and data visualization

```
col_f4e_poly2_mean$col_mock_smothed <- as.numeric(ma(col_f4e_poly2_mean$mean_col_mock, 
                                                     order = 50, centre = TRUE))
col_f4e_poly2_mean$col_tumv_smothed <- as.numeric(ma(col_f4e_poly2_mean$mean_col_tumv, 
                                                     order = 50, centre = TRUE))
col_f4e_poly2_mean$f4e_mock_smothed <- as.numeric(ma(col_f4e_poly2_mean$mean_f4e_mock, 
                                                     order = 50, centre = TRUE))
col_f4e_poly2_mean$f4e_tumv_smothed <- as.numeric(ma(col_f4e_poly2_mean$mean_f4e_tumv, 
                                                     order = 50, centre = TRUE))

# Data visualization

plot(x = XMIN : XMAX, y = col_f4e_poly2_mean[XMIN : XMAX, 5], 
     type = "l", xlim = c(XMIN, XMAX), ylim = c(YMIN, 0.1), 
     col = "seagreen", 
     xlab = "", ylab = "", lwd = 2, frame.plot = FALSE)
points(x = XMIN : XMAX, y = col_f4e_poly2_mean[XMIN : XMAX, 6], 
       type = "l", xlim = c(XMIN, XMAX), ylim = c(YMIN, 0.1),
       col = "darkgreen", lwd = 2,)
points(x = XMIN : XMAX, y = col_f4e_poly2_mean[XMIN : XMAX, 7], 
       type = "l", xlim = c(XMIN, XMAX), ylim = c(YMIN, 0.1),
       col = "gold", lwd = 2,)
points(x = XMIN : XMAX, y = col_f4e_poly2_mean[XMIN : XMAX, 8], 
       type = "l", xlim = c(XMIN, XMAX), ylim = c(YMIN, 0.1),
       col = "red", lwd = 2,)
legend("topright", legend = c("col mock", "col tumv", "eif4e mock", "eif4e tumv"),
       col = c("seagreen", "darkgreen", "gold", "red"), lty = 1, lwd = 2,
       box.lty = 0)
```

fig.8 : Smoothed polysome profiles mean of replicates

## 6.2 Normalisation of the data

We will demonstrate two methods of polysome profiling data
normalization: 1/ Normalization by monosome and 2/ Normalization by
disome (i.e. first polysome).

Normalization by monosome allows us to assess the translation level
for each condition while keeping the initiation (monosome) at the same
level.

Normalization by disome enables us to compare the initiation and
translation levels when the amount of transcript linked to two ribosomes
is identical.

### 6.2.1 Normalisation by monosome

To normalize by monosome, we first identify the minimum value before
the monosome peak and the minimum value after the monosome peak for each
curve. We then calculate the Y-value for the index mono\_pic - MEAN\_START
for each curve.

Next, we prepare the data for normalization, dividing each curve by
the corresponding Y-value to obtain the normalized data.

Finally, we plot the normalized absorbance profiles for each
condition.

```
# Determinie the index of the minimum before the monosome pic
min1_col_mock <- which(col_f4e_poly2_mean == min(col_f4e_poly2_mean[ZMONO:(mono_pic - MEAN_START), 5], 
                                                 na.rm = TRUE),  arr.ind = TRUE)[1]
min1_col_tumv <- which(col_f4e_poly2_mean == min(col_f4e_poly2_mean[ZMONO:(mono_pic - MEAN_START), 6], 
                                                 na.rm = TRUE), arr.ind = TRUE)[1]
min1_f4e_mock <- which(col_f4e_poly2_mean == min(col_f4e_poly2_mean[ZMONO:(mono_pic - MEAN_START), 7], 
                                                 na.rm = TRUE), arr.ind = TRUE)[1]
min1_f4e_tumv <- which(col_f4e_poly2_mean == min(col_f4e_poly2_mean[ZMONO:(mono_pic - MEAN_START), 8], 
                                                 na.rm = TRUE), arr.ind = TRUE)[1]

# Determinie the index of the minimum after the monosome pic
min2_col_mock <- which(col_f4e_poly2_mean == min(col_f4e_poly2_mean[(mono_pic-MEAN_START): XMAX, 5], 
                                                 na.rm = TRUE),  arr.ind = TRUE)[1]
min2_col_tumv <- which(col_f4e_poly2_mean == min(col_f4e_poly2_mean[(mono_pic-MEAN_START): XMAX, 6], 
                                                 na.rm = TRUE), arr.ind = TRUE)[1]
min2_f4e_mock <- which(col_f4e_poly2_mean == min(col_f4e_poly2_mean[(mono_pic-MEAN_START): XMAX, 7], 
                                                 na.rm = TRUE), arr.ind = TRUE)[1]
min2_f4e_tumv <- which(col_f4e_poly2_mean == min(col_f4e_poly2_mean[(mono_pic-MEAN_START): XMAX, 8], 
                                                 na.rm = TRUE), arr.ind = TRUE)[1]

# Identify Y for the index mono_pic - MEAN_START for each curve
Y_MONO_col_mock <- col_f4e_poly2_mean[mono_pic - MEAN_START,5]
Y_MONO_col_tumv <- col_f4e_poly2_mean[mono_pic - MEAN_START,6]
Y_MONO_f4e_mock <- col_f4e_poly2_mean[mono_pic - MEAN_START,7]
Y_MONO_f4e_tumv <- col_f4e_poly2_mean[mono_pic - MEAN_START,8]

# Prepare the data
mono_norm <- data.frame(c(col_f4e_poly2_mean[XMIN : XMAX, 5]/Y_MONO_col_mock), 
                        c(col_f4e_poly2_mean[XMIN : XMAX, 6]/Y_MONO_col_tumv ),
                        c(col_f4e_poly2_mean[XMIN : XMAX, 7]/Y_MONO_f4e_mock),
                        c(col_f4e_poly2_mean[XMIN : XMAX, 8]/Y_MONO_f4e_tumv))
names(mono_norm) <- c("col_mock", "col_tumv", "f4e_mock", "f4e_tumv")

# Visualize the monosome-normalized absorbance profiles. The colors, legend, and the specific experimental conditions displayed in the plot are customizable.

plot(x = XMIN : XMAX, y = (mono_norm$col_mock), 
     type = "l", xlim = c(XMIN, XMAX), ylim = c(YMIN, 1), 
     col = "green", 
     xlab = "", ylab = "", lwd = 2, cex.axis = 0.75, frame.plot = FALSE)
points(x = XMIN : XMAX, y = (mono_norm$col_tumv), 
       type = "l", xlim = c(XMIN, XMAX), ylim = c(YMIN, 1),
       col = "darkgreen", lwd = 2,)
points(x = XMIN : XMAX, y = (mono_norm$f4e_mock), 
       type = "l", xlim = c(XMIN, XMAX), ylim = c(YMIN, 1),
       col = "gold", lwd = 2,)
points(x = XMIN : XMAX, y = (mono_norm$f4e_tumv), 
       type = "l", xlim = c(XMIN, XMAX), ylim = c(YMIN, 1),
       col = "red", lwd = 2,)

legend("topright", legend = c("col mock", "col tumv", "eif4e mock", "eif4e tumv"),
       col = c("seagreen", "darkgreen", "gold", "red"), lty = 1, lwd = 2,
       box.lty = 0)
```

fig.9 : Polysome profiles normalised to monosome

### 6.2.2 Normalisation by disome

To normalize by disome, the curves need to be aligned on the disome
peak. To determine the position of the disome peak, it is necessary to
identify the maximum before the monosome peak for all curves.

```
# Identify the index of maximum in XMIN : min1_XX_XX 
diso_col_mock <- which(col_f4e_poly2_mean == max(col_f4e_poly2_mean[XMIN: min1_col_mock, 5], 
                                                 na.rm = TRUE), arr.ind = TRUE)[1]
diso_f4e_mock <- which(col_f4e_poly2_mean == max(col_f4e_poly2_mean[XMIN: min1_f4e_mock, 7], 
                                                 na.rm = TRUE), arr.ind = TRUE)[1]
diso_col_tumv <- which(col_f4e_poly2_mean == max(col_f4e_poly2_mean[XMIN: min1_col_tumv, 6], 
                                                 na.rm = TRUE), arr.ind = TRUE)[1]
diso_f4e_tumv <- which(col_f4e_poly2_mean == max(col_f4e_poly2_mean[XMIN: min1_f4e_tumv, 8], 
                                                 na.rm = TRUE), arr.ind = TRUE)[1]
diso_pic <- min(c(diso_col_mock, diso_f4e_mock, diso_col_tumv, diso_f4e_tumv))

#Identify Y for the maximum in XMIN : min1_XX_XX  
Y_diso_pic__col_mock <- max(col_f4e_poly2_mean[XMIN: min1_col_mock, 5], na.rm = TRUE)
Y_diso_pic__col_tumv <- max(col_f4e_poly2_mean[XMIN: min1_col_tumv, 6], na.rm = TRUE)
Y_diso_pic__f4e_mock <- max(col_f4e_poly2_mean[XMIN: min1_f4e_mock, 7], na.rm = TRUE)
Y_diso_pic__f4e_tumv <- max(col_f4e_poly2_mean[XMIN: min1_f4e_tumv, 8], na.rm = TRUE)

diso_norm <- data.frame(c(col_f4e_poly2_mean$col_mock_smothed[-(1 : (diso_col_mock - diso_pic))]/Y_diso_pic__col_mock)[XMIN:XMAX], 
                        c(col_f4e_poly2_mean$col_tumv_smothed[-(1 : (diso_col_tumv - diso_pic))]/Y_diso_pic__col_tumv)[XMIN:XMAX],
                        c(col_f4e_poly2_mean$f4e_mock_smothed[-(1 : (diso_f4e_mock - diso_pic))]/Y_diso_pic__f4e_mock)[XMIN:XMAX],
                        c(col_f4e_poly2_mean$f4e_tumv_smothed[-(1 : (diso_f4e_tumv - diso_pic))]/Y_diso_pic__f4e_tumv)[XMIN:XMAX])
names(diso_norm) <- c("col_mock", "col_tumv", "f4e_mock", "f4e_tumv")

# Visualize the disome-normalized absorbance profiles. The colors, legend, and the specific experimental conditions displayed in the plot are customizable.

plot(x = XMIN : XMAX, y = (diso_norm$col_mock), 
     type = "l", xlim = c(XMIN, XMAX), ylim = c(YMIN, 6), 
     col = "green", 
     xlab = "", ylab = "", lwd = 2, cex.axis = 0.75, frame.plot = FALSE)
points(x = XMIN : XMAX, y = (diso_norm$col_tumv), 
       type = "l", xlim = c(XMIN, XMAX), ylim = c(YMIN, 6),
       col = "darkgreen", lwd = 2,)
points(x = XMIN : XMAX, y = (diso_norm$f4e_mock), 
       type = "l", xlim = c(XMIN, XMAX), ylim = c(YMIN, 6),
       col = "gold", lwd = 2,)
points(x = XMIN : XMAX, y = (diso_norm$f4e_tumv), 
       type = "l", xlim = c(XMIN, XMAX), ylim = c(YMIN, 6),
       col = "red", lwd = 2,)

legend("topright", legend = c("col mock", "col tumv", "eif4e mock", "eif4e tumv"),
       col = c("seagreen", "darkgreen", "gold", "red"), lty = 1, lwd = 2,
       box.lty = 0)
```

fig.10 : Polysome profiles normalised to disome (i.e. first polysome)

# 7 References

1. Andri et mult. al., Signorell. 2021. DescTools: Tools for
   Descriptive Statistics. https://cran.r-project.org/package=DescTools.
2. Hyndman, Rob J, and Yeasmin Khandakar. 2008. “Automatic Time
   Series Forecasting: The Forecast Package for R.” Journal of Statistical
   Software 26 (3): 1–22. https://doi.org/10.18637/jss.v027.i03.
3. Kassambara, Alboukadel. 2021. Rstatix: Pipe-Friendly Framework
   for Basic Statistical Tests. https://CRAN.R-project.org/package=rstatix.
4. R Core Team. 2021. R: A Language and Environment for Statistical
   Computing. Vienna, Austria: R Foundation for Statistical Computing. https://www.R-project.org/.
5. Wickham, Hadley. 2016. Ggplot2: Elegant Graphics for Data
   Analysis. Springer-Verlag New York. https://ggplot2.tidyverse.org.
6. Wickham, Hadley. 2021. Tidyr: Tidy Messy Data. https://CRAN.R-project.org/package=tidyr.
7. Wickham, Hadley, Romain François, Lionel Henry, and Kirill
   Müller. 2021. Dplyr: A Grammar of Data Manipulation. https://CRAN.R-project.org/package=dplyr.
8. Xie, Yihui. 2014. “Knitr: A Comprehensive Tool for Reproducible
   Research in R.” In Implementing Reproducible Computational Research,
   edited by Victoria Stodden, Friedrich Leisch, and Roger D. Peng.
   Chapman; Hall/CRC. http://www.crcpress.com/product/isbn/9781466561595.
9. Xie, Yihui, Christophe Dervieux, and Emily Riederer. 2020. R
   Markdown Cookbook. Boca Raton, Florida: Chapman; Hall/CRC. https://bookdown.org/yihui/rmarkdown-cookbook.

# 8 R session information

```
InfoSession <- devtools::session_info()
sink("InfoSession.txt")
print(InfoSession)
```

```
## ─ Session info ───────────────────────────────────────────────────────────────
##  setting  value
##  version  R version 4.3.0 (2023-04-21 ucrt)
##  os       Windows 11 x64 (build 22621)
##  system   x86_64, mingw32
##  ui       RTerm
##  language (EN)
##  collate  English_United States.utf8
##  ctype    English_United States.utf8
##  tz       Europe/Paris
##  date     2023-09-06
##  pandoc   2.19.2 @ C:/Program Files/RStudio/resources/app/bin/quarto/bin/tools/ (via rmarkdown)
## 
## ─ Packages ───────────────────────────────────────────────────────────────────
##  package     * version  date (UTC) lib source
##  abind         1.4-5    2016-07-21 [1] CRAN (R 4.3.0)
##  backports     1.4.1    2021-12-13 [1] CRAN (R 4.3.0)
##  boot          1.3-28.1 2022-11-22 [2] CRAN (R 4.3.0)
##  broom         1.0.4    2023-03-11 [1] CRAN (R 4.3.0)
##  bslib         0.4.2    2022-12-16 [1] CRAN (R 4.3.0)
##  cachem        1.0.7    2023-02-24 [1] CRAN (R 4.3.0)
##  callr         3.7.3    2022-11-02 [1] CRAN (R 4.3.0)
##  car           3.1-2    2023-03-30 [1] CRAN (R 4.3.0)
##  carData       3.0-5    2022-01-06 [1] CRAN (R 4.3.0)
##  cellranger    1.1.0    2016-07-27 [1] CRAN (R 4.3.0)
##  class         7.3-21   2023-01-23 [2] CRAN (R 4.3.0)
##  cli           3.6.1    2023-03-23 [1] CRAN (R 4.3.0)
##  colorspace    2.1-0    2023-01-23 [1] CRAN (R 4.3.0)
##  crayon        1.5.2    2022-09-29 [1] CRAN (R 4.3.0)
##  curl          5.0.0    2023-01-12 [1] CRAN (R 4.3.0)
##  data.table    1.14.8   2023-02-17 [1] CRAN (R 4.3.0)
##  DescTools   * 0.99.48  2023-02-19 [1] CRAN (R 4.3.0)
##  devtools      2.4.5    2022-10-11 [1] CRAN (R 4.3.0)
##  digest        0.6.31   2022-12-11 [1] CRAN (R 4.3.0)
##  dplyr       * 1.1.2    2023-04-20 [1] CRAN (R 4.3.0)
##  e1071         1.7-13   2023-02-01 [1] CRAN (R 4.3.0)
##  ellipsis      0.3.2    2021-04-29 [1] CRAN (R 4.3.0)
##  evaluate      0.20     2023-01-17 [1] CRAN (R 4.3.0)
##  Exact         3.2      2022-09-25 [1] CRAN (R 4.3.0)
##  expm          0.999-7  2023-01-09 [1] CRAN (R 4.3.0)
##  fansi         1.0.4    2023-01-22 [1] CRAN (R 4.3.0)
##  farver        2.1.1    2022-07-06 [1] CRAN (R 4.3.0)
##  fastmap       1.1.1    2023-02-24 [1] CRAN (R 4.3.0)
##  forecast    * 8.21     2023-02-27 [1] CRAN (R 4.3.1)
##  fracdiff      1.5-2    2022-10-31 [1] CRAN (R 4.3.1)
##  fs            1.6.2    2023-04-25 [1] CRAN (R 4.3.0)
##  generics      0.1.3    2022-07-05 [1] CRAN (R 4.3.0)
##  ggplot2     * 3.4.2    2023-04-03 [1] CRAN (R 4.3.1)
##  gld           2.6.6    2022-10-23 [1] CRAN (R 4.3.0)
##  glue          1.6.2    2022-02-24 [1] CRAN (R 4.3.0)
##  gtable        0.3.3    2023-03-21 [1] CRAN (R 4.3.0)
##  highr         0.10     2022-12-22 [1] CRAN (R 4.3.0)
##  htmltools     0.5.5    2023-03-23 [1] CRAN (R 4.3.0)
##  htmlwidgets   1.6.2    2023-03-17 [1] CRAN (R 4.3.0)
##  httpuv        1.6.9    2023-02-14 [1] CRAN (R 4.3.0)
##  httr          1.4.5    2023-02-24 [1] CRAN (R 4.3.0)
##  jquerylib     0.1.4    2021-04-26 [1] CRAN (R 4.3.0)
##  jsonlite      1.8.4    2022-12-06 [1] CRAN (R 4.3.0)
##  knitr         1.42     2023-01-25 [1] CRAN (R 4.3.0)
##  labeling      0.4.2    2020-10-20 [1] CRAN (R 4.3.0)
##  later         1.3.0    2021-08-18 [1] CRAN (R 4.3.0)
##  lattice       0.21-8   2023-04-05 [2] CRAN (R 4.3.0)
##  lifecycle     1.0.3    2022-10-07 [1] CRAN (R 4.3.0)
##  lmom          2.9      2022-05-29 [1] CRAN (R 4.3.0)
##  lmtest        0.9-40   2022-03-21 [1] CRAN (R 4.3.0)
##  magrittr      2.0.3    2022-03-30 [1] CRAN (R 4.3.0)
##  MASS          7.3-58.4 2023-03-07 [2] CRAN (R 4.3.0)
##  Matrix        1.5-4    2023-04-04 [2] CRAN (R 4.3.0)
##  memoise       2.0.1    2021-11-26 [1] CRAN (R 4.3.0)
##  mime          0.12     2021-09-28 [1] CRAN (R 4.3.0)
##  miniUI        0.1.1.1  2018-05-18 [1] CRAN (R 4.3.0)
##  munsell       0.5.0    2018-06-12 [1] CRAN (R 4.3.0)
##  mvtnorm       1.1-3    2021-10-08 [1] CRAN (R 4.3.0)
##  nlme          3.1-162  2023-01-31 [2] CRAN (R 4.3.0)
##  nnet          7.3-18   2022-09-28 [2] CRAN (R 4.3.0)
##  pillar        1.9.0    2023-03-22 [1] CRAN (R 4.3.0)
##  pkgbuild      1.4.0    2022-11-27 [1] CRAN (R 4.3.0)
##  pkgconfig     2.0.3    2019-09-22 [1] CRAN (R 4.3.0)
##  pkgload       1.3.2    2022-11-16 [1] CRAN (R 4.3.0)
##  prettyunits   1.1.1    2020-01-24 [1] CRAN (R 4.3.0)
##  processx      3.8.1    2023-04-18 [1] CRAN (R 4.3.0)
##  profvis       0.3.7    2020-11-02 [1] CRAN (R 4.3.0)
##  promises      1.2.0.1  2021-02-11 [1] CRAN (R 4.3.0)
##  proxy         0.4-27   2022-06-09 [1] CRAN (R 4.3.0)
##  ps            1.7.5    2023-04-18 [1] CRAN (R 4.3.0)
##  purrr         1.0.1    2023-01-10 [1] CRAN (R 4.3.0)
##  quadprog      1.5-8    2019-11-20 [1] CRAN (R 4.3.0)
##  quantmod      0.4.24   2023-07-17 [1] CRAN (R 4.3.1)
##  R6            2.5.1    2021-08-19 [1] CRAN (R 4.3.0)
##  Rcpp          1.0.10   2023-01-22 [1] CRAN (R 4.3.0)
##  readxl        1.4.2    2023-02-09 [1] CRAN (R 4.3.0)
##  remotes       2.4.2    2021-11-30 [1] CRAN (R 4.3.0)
##  rlang         1.1.0    2023-03-14 [1] CRAN (R 4.3.0)
##  rmarkdown     2.21     2023-03-26 [1] CRAN (R 4.3.0)
##  rootSolve     1.8.2.3  2021-09-29 [1] CRAN (R 4.3.0)
##  rstatix     * 0.7.2    2023-02-01 [1] CRAN (R 4.3.0)
##  rstudioapi    0.14     2022-08-22 [1] CRAN (R 4.3.0)
##  sass          0.4.5    2023-01-24 [1] CRAN (R 4.3.0)
##  scales        1.2.1    2022-08-20 [1] CRAN (R 4.3.0)
##  sessioninfo   1.2.2    2021-12-06 [1] CRAN (R 4.3.0)
##  shiny         1.7.4    2022-12-15 [1] CRAN (R 4.3.0)
##  stringi       1.7.12   2023-01-11 [1] CRAN (R 4.3.0)
##  stringr       1.5.0    2022-12-02 [1] CRAN (R 4.3.0)
##  tibble        3.2.1    2023-03-20 [1] CRAN (R 4.3.0)
##  tidyr       * 1.3.0    2023-01-24 [1] CRAN (R 4.3.0)
##  tidyselect    1.2.0    2022-10-10 [1] CRAN (R 4.3.0)
##  timeDate      4022.108 2023-01-07 [1] CRAN (R 4.3.0)
##  tseries       0.10-54  2023-05-02 [1] CRAN (R 4.3.1)
##  TTR           0.24.3   2021-12-12 [1] CRAN (R 4.3.1)
##  urca          1.3-3    2022-08-29 [1] CRAN (R 4.3.1)
##  urlchecker    1.0.1    2021-11-30 [1] CRAN (R 4.3.0)
##  usethis       2.1.6    2022-05-25 [1] CRAN (R 4.3.0)
##  utf8          1.2.3    2023-01-31 [1] CRAN (R 4.3.0)
##  vctrs         0.6.2    2023-04-19 [1] CRAN (R 4.3.0)
##  withr         2.5.0    2022-03-03 [1] CRAN (R 4.3.0)
##  xfun          0.39     2023-04-20 [1] CRAN (R 4.3.0)
##  xtable        1.8-4    2019-04-21 [1] CRAN (R 4.3.0)
##  xts           0.13.1   2023-04-16 [1] CRAN (R 4.3.1)
##  yaml          2.3.7    2023-01-23 [1] CRAN (R 4.3.0)
##  zoo           1.8-12   2023-04-13 [1] CRAN (R 4.3.0)
## 
##  [1] C:/Users/Delyan/AppData/Local/R/win-library/4.3
##  [2] C:/Program Files/R/R-4.3.0/library
## 
## ──────────────────────────────────────────────────────────────────────────────
```

```
sink()
```
