# Supplementary material for: Arabidopsis eIF4E1 protects the translational machinery during TuMV infection and restricts virus accumulation
Source: PLoS Pathog. 2023 Nov 20;19(11):e1011417. doi: 10.1371/journal.ppat.1011417 (PMC10721207; doi:10.1371/journal.ppat.1011417)
Supplement: S1 Supporting Dataset — (ZIP) [file ppat.1011417.s010.zip › Fig 2/Fig 2a-d (MS quantification data + script)/D-23-00790_volcano plots_markdown_final.html]

Volcano plot representation of MS data


# Volcano plot representation of MS data

#### Delyan Zafirov and Kyoka Kuroiwa

#### September, 2023

---

# 1 Introduction

The analysis presented here is a component of the data described in
the article “Arabidopsis eIF4E1 protects host translation and restricts
virus accumulation during TuMV infection”. The script generates volcano
plots to visually represent mass spectrometry-based quantitative
proteomics data. This script was developed as a collaborative effort
between two individuals who engage in dedicated RStudio weekend
sessions; these sessions stimulated a productive scientific environment
but also deepened their connection, respect and passion for each other.
The initial version of the script was inspired by and adapted from the
following blog posts: https://erikaduan.github.io/posts/2021-01-02-volcano-plots-with-ggplot2/
https://www.geeksforgeeks.org/how-to-read-a-xlsx-file-with-multiple-sheets-in-r/.

# 2 Data information

The data utilized in this R script represents the relative abundance
of proteins with mRNA cap binding activity. The data was obtained
through a mass spectrometry (MS)-based quantitative proteomics approach,
following an mRNA cap analogue pull-down assay on protein extracts
obtained from wild-type (Col) and eif4e1KO (KO) plants infected (with
TuMV) or not (mock) for 21 days.

The complete mass spectrometry proteomics data has been submitted to
the ProteomeXchange Consortium via the PRIDE partner repository, with
the dataset identifier PXD036336. In this analysis, we specifically
focus on the processed mass-spectrometry data contained in the Excel
spreadsheet named ‘Volcano plots\_vf.xlsx’. This spreadsheet consists of
four different tabs representing distinct comparisons:

‘mock’: Relative protein abundance in wild-type versus eif4e1KO
mock-inoculated plants  
‘TuMV’: Wild-type TuMV-infected plants versus eif4e1 TuMV-infected
plants  
‘WT’: Wild-type mock-inoculated plants versus wild-type TuMV-infected
plants  
‘KO’: eif4e1KO mock-inoculated plants versus eif4e1 TuMV-infected
plants

# 3 Data preparation

This section prepares the data for analysis, including loading the
necessary packages, reading the data file, and adding an
‘enrichment\_type’ column to categorize proteins based on their fold
change and adjusted p-value.

```
# Install the following packages: 

if (!require("pacman")) install.packages("pacman")
```

```
## Loading required package: pacman
```

```
pacman::p_load(here,
               tidyverse,
               janitor, # Cleaning column names
               scales, # Transform axis scales
               ggrepel) # Optimise plot label separation

if (!require("readxl")) install.packages("readxl")
```

```
## Loading required package: readxl
```

```
# Define the path. The value of path should be specified to your desired directory path.

path <- "C:/Users/Delyan/Desktop/KK volcano outburst/Volcano plots_vf.xlsx"
multiplesheets <- function(fname) {
  sheets <- readxl::excel_sheets(fname)
  tibble <- lapply(sheets, function(x) readxl::read_excel(fname, sheet = x))
  data_frame <- lapply(tibble, as.data.frame)
  names(data_frame) <- sheets
  cat("") 
  invisible(data_frame)
}

# Load the data

volcanofile <-multiplesheets(path)
```

```
## New names:
## • `` -> `...6`
```

```
## New names:
## New names:
## New names:
## • `` -> `...6`
```

```
# Here we are going to work with the tab named "WT":

all_wt_Mock_vs_TuMV <- volcanofile$WT

# pick only the 3 columns we need. These are the columns 'accession' (i.e the uniprot ID of the protein), 'fold_change' (i.e the log2 fold change in protein abundance) and adj_p_value (the -log10 limma p-value).

wt_Mock_vs_TuMV <- all_wt_Mock_vs_TuMV [, c("accession", "fold_change", "adj_p_value")]


# An additional column labeled 'enrichment_type' will be incorporated into the data. Proteins with a fold_change value >= l and an adj_p_value >= 2 will be categorized as "up" or "down". Proteins that do not surpass these thresholds will be labeled as "ns" (non-significant).

wt_Mock_vs_TuMV <- wt_Mock_vs_TuMV %>%
  mutate(enrichment_type = case_when(fold_change >= 1 & -log10(adj_p_value) >= 2 ~ "up",
                               fold_change <= -1 & -log10(adj_p_value) >= 2 ~ "down",
                               TRUE ~ "ns"))

# The file now contains 4 columns
head(wt_Mock_vs_TuMV)
```

## 3.1 Labelling the proteins of interest

In this section, we specify the proteins of interest that we wish to
highlight in the volcano plot. In our case, these are the 5 main
translation initiation factors 4E and 4G in Arabidopsis - ‘eIF4E1’,
‘eIF4G’, ‘eIFiso4G1’, ‘eIFiso4G2’, ‘eIFiso4E’.

```
# Select the first five rows from the data frame 'wt_Mock_vs_TuMV' and create a new data frame 'poi'. <br> 'poi' stands for 'proteins of interest' and encompasses the translation initiation factors 4E and 4G  that we want to highlight in the volcano plot. Note that the proteins of interest in this case have been manually moved up in the excel spreadsheet to facilitate pinpointing them.
poi <- wt_Mock_vs_TuMV %>% slice(c(1:5),) %>% print()
```

```
##     accession fold_change adj_p_value enrichment_type
## 1 IF4E1_ARATH   0.8609627  0.14602045              ns
## 2  IF4G_ARATH   1.6525492  0.01106312              ns
## 3 IF4G1_ARATH   1.6848544  0.03235935              ns
## 4 IF4G2_ARATH   1.7366297  0.02383803              ns
## 5 IF4E4_ARATH   0.9154212  0.22463088              ns
```

```
# Create a character vector 'poi_id' containing specific values. Here we modify the protein names according to the standard naming convention for plant translation initiation factors. Nevertheless, it is possible to customize this part to match the particular name of the protein of interest:
poi_id <- c('eIF4E1', 'eIF4G', 'eIFiso4G1', 'eIFiso4G2', 'eIFiso4E')

# Add a new column 'id' to the 'poi' data frame and assign 'poi_id' values to it:
poi$id <- poi_id

# Convert the 'id' column to a character vector:
poi$id <- as.character(poi$id)

# Convert the 'enrichment_type' column to a factor:
poi$enrichment_type <- as.factor(poi$enrichment_type)

# Create a new data frame 'up_poi' containing rows where 'enrichment_type' is "up":
up_poi <- poi %>% filter(enrichment_type == "up")

# Create a new data frame 'down_poi' containing rows where 'enrichment_type' is "down":
down_poi <- poi %>% filter(enrichment_type == "down")

# Create a new data frame 'ns_poi' containing rows where 'enrichment_type' is "ns":
ns_poi <- poi %>% filter(enrichment_type == "ns")
```

# 4 Data visualization: wild-type mock vs wild-type TuMV

In this section, we will create the volcano plot to visualize the
relative abundance of cap-binding proteins between wild-type mock and
wild-type TuMV samples, with translation initiation factors 4E and 4G
highlighted.

```
wt_Mock_vs_TuMV$enrichment_type <- as.factor(wt_Mock_vs_TuMV$enrichment_type)
wt_Mock_vs_TuMV <- wt_Mock_vs_TuMV %>%
  mutate(enrichment_type = fct_relevel(enrichment_type, "up", "down","ns"))

# Define colors, sizes, and transparencies for different enrichment types:
cols <- c("up" = "mediumseagreen", "down" = "magenta4", "ns" = "grey")
sizes <- c("up" = 3, "down" = 3, "ns" = 2)
alphas <- c("up" = 1, "down" = 1, "ns" = 0.1)

# Generate the volcano plot using ggplot:
wt_Mock_vs_TuMV %>%
  ggplot(aes(x = fold_change,
             y = -log10(adj_p_value)))+
  geom_point(aes(color = enrichment_type), shape = 19, size = 3, alpha = 0.5) +
  scale_color_manual(values = cols)+ # Modify point colour
  geom_hline(yintercept = 2,
             linetype = "dashed",
             color="grey") +
  geom_vline(xintercept = c(-1, 1),
             linetype = "dashed",
             color="grey") +
  scale_x_continuous(breaks = c(seq(-8, 10, 2)), # X-axis tick intervals can me modified
                     limits = c(-8, 10.5))+
  scale_y_continuous(breaks = c(seq(0, 10, 2)), # Y-axis tick intervals can me modified
                     limits = c(0, 10)) +
  labs(title="wild-type mock vs wild-type TuMV") +
  theme_classic() +
  theme(legend.position = "none")+
  geom_point(data = up_poi, # New layer containing a protein of interest data subset
             size = 4,
             shape = 21,
             alpha = 1,
             fill = "darkgreen",
             colour = "black") +
  geom_point(data = ns_poi, # New layer containing a protein of interest data subset
             size = 4,
             shape = 21,
             alpha = 1,
             fill = "grey50",
             colour = "black") +
  geom_point(data = down_poi, # New layer containing a protein of interest data subset, note that nudge_y =3 can be opted out if the labels of the poi are not fully shown
             size = 4,
             shape = 21,
             alpha = 1,
             fill = "purple",
             colour = "black") +
  geom_text_repel(data = poi,
                  aes(label = id),
                  force_pull = 4,
                  nudge_y = 3,
                  segment.size = 0.1,
                  box.padding = 4,
                  point.padding = 0,
                  color = "black",
                  direction = "x",
                  size= 3.5)
```

MS-based quantitative comparison of protein abundance: **wild-type
mock vs wild-type TuMV**

# 5 Data visualization: Wild-type TuMV vs wild-type mock

The previous volcano plot displayed the relative abundance of
proteins in wild-type mock compared to wild-type TuMV. Now, we will
reverse the comparison to visualize the data as wild-type TuMV versus
wild-type mock. The following script presents the new volcano plot for
this inverted representation.

```
# Make a copy of the original dataframe
wt_TuMV_vs_mock <- wt_Mock_vs_TuMV

# To reverse the comparison from wild-type mock versus wild-type TuMV to wild-type TuMV versus wild-type mock, it is only necessary to invert the sign of the values in the 'fold_change' column. As the fold_change values are expressed in log2 format, inverting these values will automatically reverse the direction of the comparisons (wild-type TuMV over wild-type mock instead of wild-type mock over wild-type TuMV).

wt_TuMV_vs_mock$fold_change <- wt_TuMV_vs_mock$fold_change * -1

# Repeat the procedure of selecting the proteins of interest as described above (point 3.1):

rev_poi <- wt_TuMV_vs_mock %>% slice(c(1:5),) %>% print()
```

```
##     accession fold_change adj_p_value enrichment_type
## 1 IF4E1_ARATH  -0.8609627  0.14602045              ns
## 2  IF4G_ARATH  -1.6525492  0.01106312              ns
## 3 IF4G1_ARATH  -1.6848544  0.03235935              ns
## 4 IF4G2_ARATH  -1.7366297  0.02383803              ns
## 5 IF4E4_ARATH  -0.9154212  0.22463088              ns
```

```
rev_poi_id <- c('eIF4E1', 'eIF4G', 'eIFiso4G1', 'eIFiso4G2', 'eIFiso4E')
rev_poi$id <- rev_poi_id
rev_poi$id <- as.character(rev_poi$id)
rev_poi$enrichment_type <- as.factor(rev_poi$enrichment_type)
up_rev_poi <- rev_poi %>% filter(enrichment_type == "up")
down_rev_poi <- rev_poi %>% filter(enrichment_type == "down")
ns_rev_poi <- rev_poi %>% filter(enrichment_type == "ns")

# Procede with data visualization as described above (rev_point 4)

wt_TuMV_vs_mock$enrichment_type <- as.factor(wt_TuMV_vs_mock$enrichment_type)
wt_TuMV_vs_mock <- wt_TuMV_vs_mock %>%
  mutate(enrichment_type = fct_relevel(enrichment_type, "up", "down","ns"))

# Define colors, sizes, and transparencies for different enrichment types:
cols <- c("up" = "mediumseagreen", "down" = "magenta4", "ns" = "grey")
sizes <- c("up" = 3, "down" = 3, "ns" = 2)
alphas <- c("up" = 1, "down" = 1, "ns" = 0.1)

# Generate the volcano plot using ggplot:
wt_TuMV_vs_mock %>%
  ggplot(aes(x = fold_change,
             y = -log10(adj_p_value)))+
  geom_point(aes(color = enrichment_type), shape = 19, size = 3, alpha = 0.5) +
  scale_color_manual(values = cols)+ # Modify rev_point colour
  geom_hline(yintercept = 2,
             linetype = "dashed",
             color="grey") +
  geom_vline(xintercept = c(-1, 1),
             linetype = "dashed",
             color="grey") +
  scale_x_continuous(breaks = c(seq(-8, 10, 2)), # X-axis tick intervals can me modified
                     limits = c(-8, 10.5))+
  scale_y_continuous(breaks = c(seq(0, 10, 2)), # Y-axis tick intervals can me modified
                     limits = c(0, 10)) +
  labs(title="wild-type TuMV vs wild-type mock") +
  theme_classic() +
  theme(legend.position = "none")+
  geom_point(data = up_rev_poi, # New layer containing a protein of interest data subset
             size = 4,
             shape = 21,
             alpha = 1,
             fill = "darkgreen",
             colour = "black") +
  geom_point(data = ns_rev_poi, # New layer containing a protein of interest data subset
             size = 4,
             shape = 21,
             alpha = 1,
             fill = "grey50",
             colour = "black") +
  geom_point(data = down_rev_poi, # New layer containing a protein of interest data subset, note that nudge_y =3 can be opted out if the labels of the poi are not fully shown
             size = 4,
             shape = 21,
             alpha = 1,
             fill = "purple",
             colour = "black") +
  geom_text_repel(data = rev_poi,
                  aes(label = id),
                  force_pull = 4,
                  nudge_y = 3,
                  segment.size = 0.1,
                  box.padding = 4,
                  rev_point.padding = 0,
                  color = "black",
                  direction = "x",
                  size= 3.5)
```

MS-based quantitative comparison of protein abundance: **wild-type
TuMV vs wild-type mock**

# 6 R session information

```
InfoSession <- devtools::session_info()
print(InfoSession)
```

```
## ─ Session info ───────────────────────────────────────────────────────────────
##  setting  value
##  version  R version 4.3.0 (2023-04-21 ucrt)
##  os       Windows 11 x64 (build 22621)
##  system   x86_64, mingw32
##  ui       RTerm
##  language (EN)
##  collate  English_United States.utf8
##  ctype    English_United States.utf8
##  tz       Europe/Paris
##  date     2023-09-06
##  pandoc   2.19.2 @ C:/Program Files/RStudio/resources/app/bin/quarto/bin/tools/ (via rmarkdown)
## 
## ─ Packages ───────────────────────────────────────────────────────────────────
##  package     * version date (UTC) lib source
##  bslib         0.4.2   2022-12-16 [1] CRAN (R 4.3.0)
##  cachem        1.0.7   2023-02-24 [1] CRAN (R 4.3.0)
##  callr         3.7.3   2022-11-02 [1] CRAN (R 4.3.0)
##  cellranger    1.1.0   2016-07-27 [1] CRAN (R 4.3.0)
##  cli           3.6.1   2023-03-23 [1] CRAN (R 4.3.0)
##  colorspace    2.1-0   2023-01-23 [1] CRAN (R 4.3.0)
##  crayon        1.5.2   2022-09-29 [1] CRAN (R 4.3.0)
##  devtools      2.4.5   2022-10-11 [1] CRAN (R 4.3.0)
##  digest        0.6.31  2022-12-11 [1] CRAN (R 4.3.0)
##  dplyr       * 1.1.2   2023-04-20 [1] CRAN (R 4.3.0)
##  ellipsis      0.3.2   2021-04-29 [1] CRAN (R 4.3.0)
##  evaluate      0.20    2023-01-17 [1] CRAN (R 4.3.0)
##  fansi         1.0.4   2023-01-22 [1] CRAN (R 4.3.0)
##  farver        2.1.1   2022-07-06 [1] CRAN (R 4.3.0)
##  fastmap       1.1.1   2023-02-24 [1] CRAN (R 4.3.0)
##  forcats     * 1.0.0   2023-01-29 [1] CRAN (R 4.3.0)
##  fs            1.6.2   2023-04-25 [1] CRAN (R 4.3.0)
##  generics      0.1.3   2022-07-05 [1] CRAN (R 4.3.0)
##  ggplot2     * 3.4.2   2023-04-03 [1] CRAN (R 4.3.1)
##  ggrepel     * 0.9.3   2023-02-03 [1] CRAN (R 4.3.0)
##  glue          1.6.2   2022-02-24 [1] CRAN (R 4.3.0)
##  gtable        0.3.3   2023-03-21 [1] CRAN (R 4.3.0)
##  here        * 1.0.1   2020-12-13 [1] CRAN (R 4.3.0)
##  highr         0.10    2022-12-22 [1] CRAN (R 4.3.0)
##  hms           1.1.3   2023-03-21 [1] CRAN (R 4.3.0)
##  htmltools     0.5.5   2023-03-23 [1] CRAN (R 4.3.0)
##  htmlwidgets   1.6.2   2023-03-17 [1] CRAN (R 4.3.0)
##  httpuv        1.6.9   2023-02-14 [1] CRAN (R 4.3.0)
##  janitor     * 2.2.0   2023-02-02 [1] CRAN (R 4.3.0)
##  jquerylib     0.1.4   2021-04-26 [1] CRAN (R 4.3.0)
##  jsonlite      1.8.4   2022-12-06 [1] CRAN (R 4.3.0)
##  knitr         1.42    2023-01-25 [1] CRAN (R 4.3.0)
##  later         1.3.0   2021-08-18 [1] CRAN (R 4.3.0)
##  lifecycle     1.0.3   2022-10-07 [1] CRAN (R 4.3.0)
##  lubridate   * 1.9.2   2023-02-10 [1] CRAN (R 4.3.0)
##  magrittr      2.0.3   2022-03-30 [1] CRAN (R 4.3.0)
##  memoise       2.0.1   2021-11-26 [1] CRAN (R 4.3.0)
##  mime          0.12    2021-09-28 [1] CRAN (R 4.3.0)
##  miniUI        0.1.1.1 2018-05-18 [1] CRAN (R 4.3.0)
##  munsell       0.5.0   2018-06-12 [1] CRAN (R 4.3.0)
##  pacman      * 0.5.1   2019-03-11 [1] CRAN (R 4.3.0)
##  pillar        1.9.0   2023-03-22 [1] CRAN (R 4.3.0)
##  pkgbuild      1.4.0   2022-11-27 [1] CRAN (R 4.3.0)
##  pkgconfig     2.0.3   2019-09-22 [1] CRAN (R 4.3.0)
##  pkgload       1.3.2   2022-11-16 [1] CRAN (R 4.3.0)
##  prettyunits   1.1.1   2020-01-24 [1] CRAN (R 4.3.0)
##  processx      3.8.1   2023-04-18 [1] CRAN (R 4.3.0)
##  profvis       0.3.7   2020-11-02 [1] CRAN (R 4.3.0)
##  promises      1.2.0.1 2021-02-11 [1] CRAN (R 4.3.0)
##  ps            1.7.5   2023-04-18 [1] CRAN (R 4.3.0)
##  purrr       * 1.0.1   2023-01-10 [1] CRAN (R 4.3.0)
##  R6            2.5.1   2021-08-19 [1] CRAN (R 4.3.0)
##  Rcpp          1.0.10  2023-01-22 [1] CRAN (R 4.3.0)
##  readr       * 2.1.4   2023-02-10 [1] CRAN (R 4.3.0)
##  readxl      * 1.4.2   2023-02-09 [1] CRAN (R 4.3.0)
##  remotes       2.4.2   2021-11-30 [1] CRAN (R 4.3.0)
##  rlang         1.1.0   2023-03-14 [1] CRAN (R 4.3.0)
##  rmarkdown     2.21    2023-03-26 [1] CRAN (R 4.3.0)
##  rprojroot     2.0.3   2022-04-02 [1] CRAN (R 4.3.0)
##  rstudioapi    0.14    2022-08-22 [1] CRAN (R 4.3.0)
##  sass          0.4.5   2023-01-24 [1] CRAN (R 4.3.0)
##  scales      * 1.2.1   2022-08-20 [1] CRAN (R 4.3.0)
##  sessioninfo   1.2.2   2021-12-06 [1] CRAN (R 4.3.0)
##  shiny         1.7.4   2022-12-15 [1] CRAN (R 4.3.0)
##  snakecase     0.11.0  2019-05-25 [1] CRAN (R 4.3.0)
##  stringi       1.7.12  2023-01-11 [1] CRAN (R 4.3.0)
##  stringr     * 1.5.0   2022-12-02 [1] CRAN (R 4.3.0)
##  tibble      * 3.2.1   2023-03-20 [1] CRAN (R 4.3.0)
##  tidyr       * 1.3.0   2023-01-24 [1] CRAN (R 4.3.0)
##  tidyselect    1.2.0   2022-10-10 [1] CRAN (R 4.3.0)
##  tidyverse   * 2.0.0   2023-02-22 [1] CRAN (R 4.3.0)
##  timechange    0.2.0   2023-01-11 [1] CRAN (R 4.3.0)
##  tzdb          0.3.0   2022-03-28 [1] CRAN (R 4.3.0)
##  urlchecker    1.0.1   2021-11-30 [1] CRAN (R 4.3.0)
##  usethis       2.1.6   2022-05-25 [1] CRAN (R 4.3.0)
##  utf8          1.2.3   2023-01-31 [1] CRAN (R 4.3.0)
##  vctrs         0.6.2   2023-04-19 [1] CRAN (R 4.3.0)
##  withr         2.5.0   2022-03-03 [1] CRAN (R 4.3.0)
##  xfun          0.39    2023-04-20 [1] CRAN (R 4.3.0)
##  xtable        1.8-4   2019-04-21 [1] CRAN (R 4.3.0)
##  yaml          2.3.7   2023-01-23 [1] CRAN (R 4.3.0)
## 
##  [1] C:/Users/Delyan/AppData/Local/R/win-library/4.3
##  [2] C:/Program Files/R/R-4.3.0/library
## 
## ──────────────────────────────────────────────────────────────────────────────
```
