# Supplementary material for: Arabidopsis eIF4E1 protects the translational machinery during TuMV infection and restricts virus accumulation
Source: PLoS Pathog. 2023 Nov 20;19(11):e1011417. doi: 10.1371/journal.ppat.1011417 (PMC10721207; doi:10.1371/journal.ppat.1011417)
Supplement: S1 Supporting Dataset — (ZIP) [file ppat.1011417.s010.zip › S2 Fig/S2 Fig unprocessed western blot.pptx]

## Slide 1
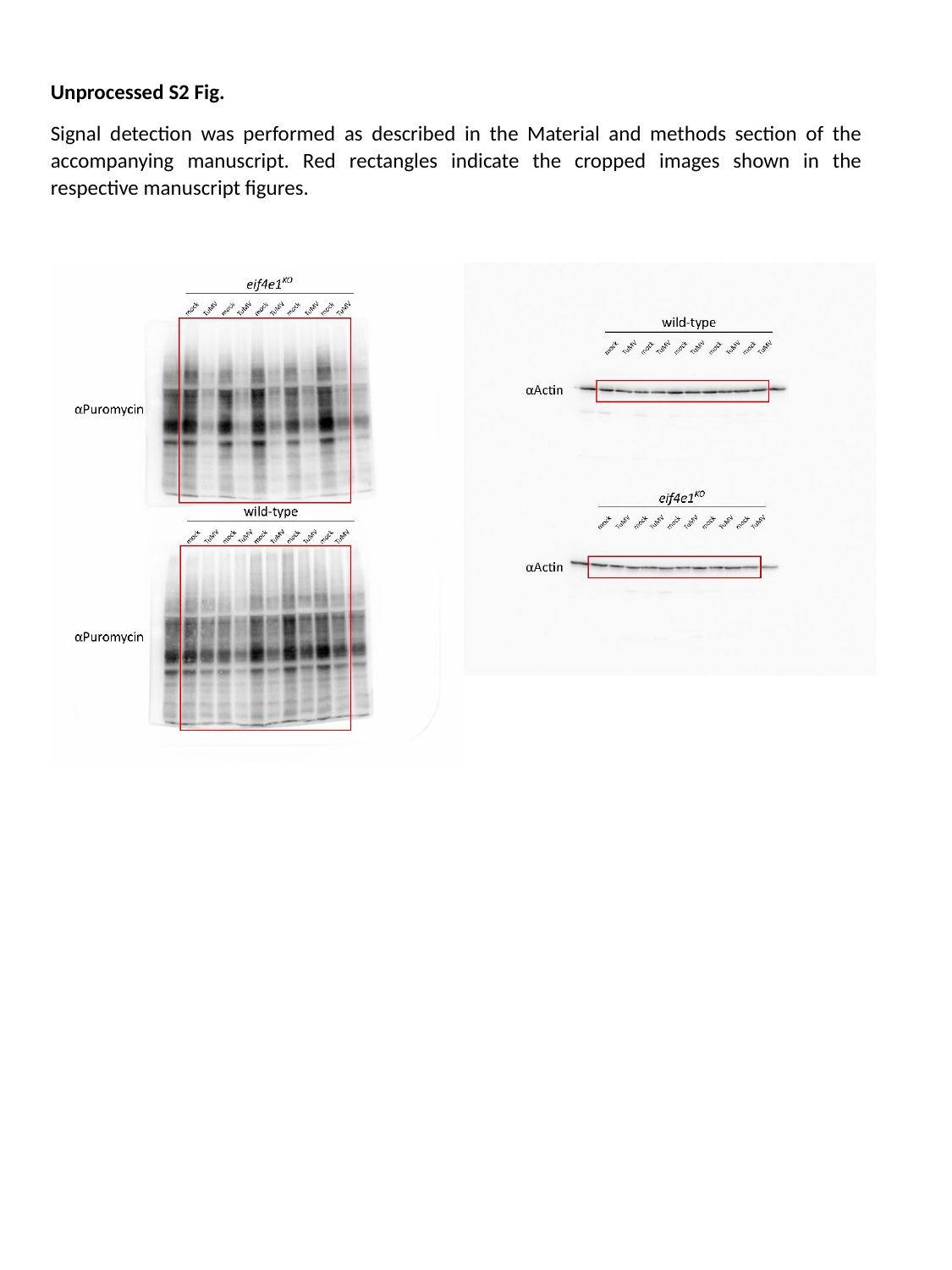

Unprocessed S2 Fig.
Signal detection was performed as described in the Material and methods section of the accompanying manuscript. Red rectangles indicate the cropped images shown in the respective manuscript figures.
