# Supplementary material for: Arabidopsis eIF4E1 protects the translational machinery during TuMV infection and restricts virus accumulation
Source: PLoS Pathog. 2023 Nov 20;19(11):e1011417. doi: 10.1371/journal.ppat.1011417 (PMC10721207; doi:10.1371/journal.ppat.1011417)
Supplement: S1 Supporting Dataset — (ZIP) [file ppat.1011417.s010.zip › S5 Fig/S5 Fig unprocessed western blot.pptx]

## Slide 1
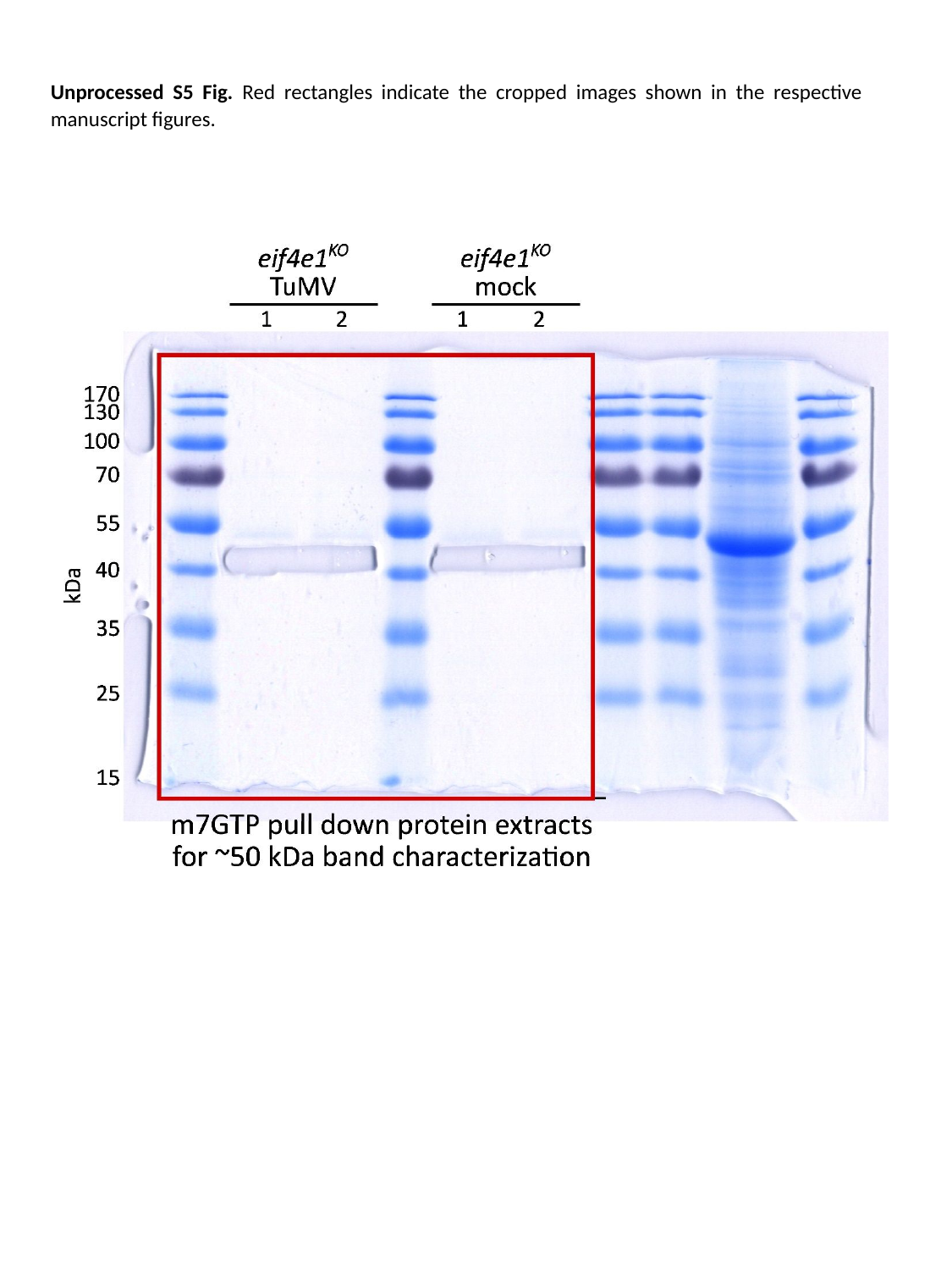

Unprocessed S5 Fig. Red rectangles indicate the cropped images shown in the respective manuscript figures.
