# Supplementary material for: Arabidopsis eIF4E1 protects the translational machinery during TuMV infection and restricts virus accumulation
Source: PLoS Pathog. 2023 Nov 20;19(11):e1011417. doi: 10.1371/journal.ppat.1011417 (PMC10721207; doi:10.1371/journal.ppat.1011417)
Supplement: Attachment — Submitted filename: PPATHOGENS-D-23-00790_Referees_answers_11h02.docx [file ppat.1011417.s011.docx]

PPATHOGENS-D-23-00790

Dear editor,

We appreciate your feedback. We are happy to provide a revised manuscript in which we address your suggestions and answer the reviewer’s questions point by point.

Best regards,

***Editor’s comments***

Overall, we found that your study provides important new insight into the potyvirus-host interaction at translation initiation complexes, with the indication of cleavage of eIFiso4G1 during infection in eIF4E mutants as a highlight in terms of novelty. In your revised version, we would especially like you pay attention to

- change the title so that it more efficiently draws the attention towards the concrete advances offered by your study

Thanks for the suggestion, we propose the following title as a more specific alternative:

“Arabidopsis *eIF4E1* protects the translational machinery during TuMV infection and restricts virus accumulation”.

- include the volcano plots currently in Figure S4 in a main figure

All volcano plots are now provided in Figure 2

- discuss more explicitly possible models to explain the low TuMV titers and exacerbated symptoms observed in eIF4E eIFiso4G1 mutants.

Yes, thank you for bringing up this matter, which is a good opportunity to discuss a topic that we find very intriguing – the interconnection between virus accumulation and symptom severity. We have added an extra paragraph at the end of the discussion (line 485 and followings) to discuss those issues:

Our investigation into single and combined Arabidopsis mutants for eIF4E1 and eIFiso4G1 has helped us address the connection between translation initiation proteins, virus accumulation, and disease symptoms in plants infected with TuMV. In particular, the simultaneous disruption of both eIF4E1 and eIFiso4G1 in the cum1 eifiso4g1KO mutant resulted in a striking reduction in growth and tissue senescence, often culminating in the collapse of the entire plant upon infection. Intriguingly, these pathological effects were not accompanied by significant viral overaccumulation, as only a limited TuMV population was detected in the living tissues of these plants. This raises a perplexing question: how can the presence of a modest amount of TuMV in cum1 eifiso4g1KO mutants lead to such pronounced disease symptoms? An appealing hypothesis is that TuMV could induce the degradation of the only remaining eIFiso4G2 protein in these plants, essentially leading to a complete breakdown of host functions due to the absence of canonical translation initiation complexes. Although eIFiso4G1 and eIFiso4G2 have different expression patterns, they share significant sequence similarities and functional overlap (Lellis et al., 2019; Bi et al., Cho et al., 2019; Cairo et al., 2022), suggesting that the mechanism responsible for eIFiso4G1 degradation might also apply to eIFiso4G2. Alternatively, and not exclusively, we could speculate that the simultaneous inactivation of eIF4E1 and eIFiso4G1 proteins might disrupt the balance of host defense and immune response pathways. The abnormal initiation complexes formed in the absence of eIF4E1 and eIFiso4G1 could influence the translation of specific host factors that modulate disease symptom development, even if the virus titers remain low. To explore this, detailed proteomic and transcriptomic analyses are needed, comparing gene expression in plants with different combinations of inactivated initiation factors. A more focused approach could involve directly monitoring the production of proteins known to influence the severity of TuMV symptoms (Butković et al., 2021; Martínez et al., 2023).

Note that the addition of this supplementary paragraph involved the addition of a couple of references which we have updated in the amended in the References section. Additionally, we have taken this opportunity to incorporate an extra reference in Line 54 of a recently published paper discussing the impact of virus infection on host translation in animal models.

***Reviewer #1’s comments***

**Part III – Minor Issues: Editorial and Data Presentation Modifications**
Please use this section for editorial suggestions as well as relatively minor modifications of existing data that would enhance clarity.

Reviewer #1: This is an interesting and well-conceived continuation of the work reported in Zafirov et al. (2021). I found particularly stimulating the observation of the truncation of eIFiso4G1 in TuMV-infected eif4e1 mutant plants. Are there alternatives to post-translational processing to explain the appearance of the eIF4iso4G1 truncated protein? Have you looked at other stress situations different than TuMV infection, for instance, heat stress?

We appreciate Reviewer #1's feedback. The exact process behind eIFiso4G1 truncation remains uncertain and captures our interest for further study. In line with the Discussion section of our manuscript (lines 438-450), we suggest that the simplest explanation for truncated eIFiso4G1 should occur at the post-translational level – possibly involving plant or viral proteases,

While our focus centers on these primary hypotheses, we acknowledge alternative scenarios leading to truncated eIFiso4G1. These could be changes in transcription (i.e. changes in transcription or splicing patterns that result in the production of an alternatively spliced or transcribed mRNA, leading to the generation of a truncated protein). Additional, unconventional translation initiation (i.e. translation start at non-canonical sites) might also yield shorter protein forms.

However, these alternatives are speculative and we currently prioritize exploring our main post-translational processing hypotheses. Nevertheless, we remain open to exploring these possibilities in the future. As for different stress conditions, we haven't investigated those (particularly regarding heat stress, please refer to our response below for Lines 414-415). Previous studies highlighting the role of eIFiso4G in abiotic stresses (Cho et al., 2019 New Phytol ; Bi et al., 2019 New Phytol) do not mention any possible cleavage, but we agree it is a line of investigation that is worth looking at in future studies.

In light of these points, we have added a phrase in our discussion section (line 446) recognizing that mechanisms alternative to protein cleavage can lead to the production of a truncated eIFiso4G1, thank you for your valuable input:

“While we propose that the proteolytic processing of eIFiso4G1 is the probable event causing the production of a truncated protein form, it is also possible that alternative mechanisms operating at the transcriptional level (such as changes in transcription or splicing patterns) or even unconventional translation initiation (like translation initiation at non-canonical sites) could also give rise to shorter eIFiso4G1 protein variants.”

In general, I found the manuscript well written and well-illustrated. Here there are a number of comments and suggestions that you may be willing to consider:

Title: I found the title rather vague, please try to be more specific. In fact, your current title may apply to several of the papers already published in the subject.

Thanks, we have proposed the following title that is more descriptive of the work presented in this manuscript: “Arabidopsis *eIF4E1* protects the translational machinery during TuMV infection and restricts virus accumulation”.

Abstract:
You use 4E/4G and eIF4E/eIF4G indistinctively. I would use only the eIF4E/eIF4G notation.

We agree this denomination can be confusing to the reader. Actually, there is a difference but it is true we did not explain it from the start. We use eIF4E/eIF4G for the actual eIF4E/eIF4G but we use 4E/4G (or 4F) to both refer to eIF and eIF isoforms. The correct replacement for 4E and 4G would be respectively eIF4E/eIFiso4E and eIF4G/eIF4iso4G (or eIF4G/eIFiso4G1/eIFiso4G2), which we find a bit heavy (but this could be done). We have now added a sentence to clarify our notation from the start.

In the abstract, we replace 4E and 4G by eIF4E, which actually can fit very well

On line 83 we added the following phrase “Throughout the text we refer to the eIF4F and eIFiso4F complexes as 4F. We use "4E" to denominate eIF4E/eIFiso4E subunits and "4G" for the eIF4G and eIFiso4G subunits.”

We also re-specified on lines 220 and following

the abundance of 4E (eIF4E1 and eIFiso4E) and 4G (eIF4G, eIFiso4G1 and eIFiso4G2) proteins were not significantly different between mock and TuMV-infected plants

Line 20: Delete called

Deleted, thanks.

Lines 24-25. I´m not sure that you provided sufficient evidence to conclude that “eIF4E1 plays crucial role in inhibiting the TuMV-induced degradation of the translation initiation factor eIFiso4G1”. I did not see any experiment showing an inhibitory effect of eIF4E1 over eIFiso4G1 degradation. In fact, I also doubt about the term degradation. Please, consider toning down this statement.

Thank you for your feedback. We have revised the statement to read as follows:

“Furthermore, our findings show that, in the absence of eIF4E1, infection with TuMV leads to the production of a truncated eIFiso4G1 protein.”

Results:
Lines 127-128. First subheading: The heading overinterprets the results and biases the reader. Please try to find a heading that reflects more precisely the results in this part.

We propose the following heading for our first results section:

The absence of eIF4E1 leads to an increase in TuMV protein synthesis while overall plant protein synthesis is reduced.

Lines 148-152. I was educated in the understanding that differences that were not statistically significant were not differences. There is an effect on mutant plants, but not in WT, though a tendency can be perceived. Please re-phrase.

We agree with the reviewer, the tendency is very tempting, but the statistics are against it if we choose as we did a 95% cutoff. We rephrased this paragraph to concur with this conclusion. A subtle effect may exit, but we would need more repetitions to see that. We now refocused this paragraph on the significant translation decrease of TuMV infection in the absence of eIF4E1.

This paragraph now reads:

In wild-type plants, the average polysome/monosome ratio was lower by 26% during TuMV infection, although the difference with the mock-inoculated control was not significant (Tukey’s HSD test, *P* = 0.1450) **(S1b, c Fig)**. In contrast, in the absence of *eIF4E1,* TuMV infection induced a significant 38% decrease in the polysome/monosome ratio compared to mock-inoculated plants (Tukey’s HSD test, *P* = 0.0282) **(Fig 1d)**. This was characterized by lower polysome levels and increased monosome accumulation, signatures of a translational impediment [52–54] **(Fig 1c, blue profiles, S1d Fig)**. Overall, more experiments would be needed to confirm whether TuMV causes a decrease in translation activity in wild-type plants, however this effect is very clear in the absence of *eIF4E1*.

Line 173. Coat protein.

Fixed, thank you.

Line178. Fig. 1 Title: “at the expense”. Why at the expense?

Well, you are right, this title is based on the rationale that because general translation decreases while viral translation increased, it should go with a decrease of host translation. But it is true that this will need more specific experiments to back this. We therefore rephrase as

“In the absence of eIF4E1, global translation activity decreases while virus accumulation increases”

Thanks.

Line 192. S1 Fig. Is this figure really providing information supplementary to that provided in Fig. 1? Please check the journal policy on what to include as Supplementary material.

We think that this figure helps to highlight differences by focusing on comparing only two experimental conditions at the same time. Our preference is to keep it, but depending on the reviewer’s and editor’s opinion, this can be removed.

Line 202. Panel C in S3 Fig. Is it the same as in Fig. 1e? If so, I do not see the need to include it again in S3 Fig.

You are right, the idea was to present all the repetitions on the same figure, hence the duplication. We have now removed panel C from S3 Fig to avoid this repetition, thanks! For more clarity, we added in S3 Fig. legend: ”The photograph of the western blot associated with the third experimental repetition is displayed in Fig 1e.”.

Lines 215-218 and results following this experiment. Would VPg in extracts from infected plants compete with the mRNA cap in the pull-downs, masking results? Please provide arguments.

Thank you for raising this interesting point about the potential interference of TuMV VPg with our M7GTP pull-down assay and the subsequent proteomics-based quantification of translation initiation factors. It has indeed been proposed that potyvirus (and in particular TuMV) VPg interacts with 4E proteins (eIFiso4E in the case of TuMV) in close vicinity to the cap-binding pocket (Khan et al. 2006, 2008; Miyoshi et al. 2008). More recent structural and biochemical data further supports the notion that potyvirus VPg may obstruct the cap-binding pocket of 4E proteins (Coutinho de Oliveira et al. 2019)

Given these findings and considering TuMV's overaccumulation in the absence of eIF4E1, it is indeed plausible that TuMV VPg could compete with eIFiso4E for m7GTP analogue binding in our pull-down assay. According to data coming from our previously published paper, we do not have any evidence for change in the abundance of eIFiso4E pulled down from wild-type and eif4e1KO mutants plants (Zafirov et al. 2021). This would suggest that there is no competition effect between TuMV VPg and the cap analogue.

Lines 220-223. Please rephrase. If differences are not statistically significant, then there are no differences.

Sure, this has been done:

In wild-type plants, the abundance of 4E (eIF4E1 and eIFiso4E) and 4G (eIF4G, eIFiso4G1 and eIFiso4G2) proteins was not significantly different between mock and TuMV-infected plants. **(Fig 2a)**.

Lines 235-236. “the unavailability of eIF4F complexes required for protein synthesis” Add “during infection”.

Fixed, thank you.

Line 247. Please consider including the S4 Fig. volcano plots in the main figure, they are very informative.

All Volcano plots are now displayed in Figure 2

Lines 264-266. But the number of counts is very low. Indeed, the ratio peptides/counts is very similar for WT and mutant (infected). Please provide convincing arguments.

We agree with the reviewer that the ratio peptides / spectral counts identified for eIFiso4G1 is similar in the different samples analyzed. However, the results that really surprised us is that we only identified peptides present in the N-terminal part of the protein in the KO-TuMV samples. In contrast, in the three other samples, peptides spanning the whole sequence of the protein were identified. This can be exemplified by looking at the FMVQNIIDMR peptide (aa 429-438) that was identified the most times in the eIFiso4G1 protein along the different samples: whereas it was identified 17 times in WT-Mock, 15 times in KO-Mock and 13 times in WT-TuMV, it was never identified in KO-TuMV (as represented in respectively Fig. 3 A, B, C and D and supplemental table). Conversely, when looking at peptides present in the N-terminal part of the protein, we can observe that there is no major differences in identification efficiency between the different samples; thus, the ETAAELFGEEGTWAR peptide (aa 96-100 of eIFiso4G1), the second most identified peptide for eIFiso4G1, was identified 7 times in WT-Mock, 5 times in KO-Mock, 7 times in WT-TuMV and 4 times in KO-TuMV. This obvious discrepancy in identification efficiency in the different samples between peptides present in the N-terminal part of eIFiso4G1 and peptides lying in the rest of the protein was striking and led us to hypothesize that eIFiso4G1 may be cleaved in KO-TuMV samples, giving rise to a truncated eIFiso4G1 containing only the N-terminal part of the protein.

We apologize if this result was not presented clearly in our manuscript. We modified the corresponding sentence and legend to Fig.3 to make it clearer. We also corrected the number of spectral counts in figure 3c from 73 to 74, a minor error from the initial analysis.

Line 269. Arabidopsis.

Corrected, thanks

Discussion:
Fig. 5. The diagram is nice, but I´m not sure that it is much needed; the ideas behind are rather simple, no need of a diagram to understand them.

Figure 5 has now been removed.

*Lines 414-415. Up to what point this response is virus-specific? Any information on what may happen during abiotic stress, for instance, heat stress?*

Thank you for your question. We don't have a direct answer, but results from the literature suggests that there may be connections. For example, under heat shock conditions: 1/ cap-dependent translation is inhibited (Wu et al., 2023 and references therein), 2/ eIFiso4G protein levels rise with unchanged mRNA levels (observation coming from wheat) (Gallie et al. 1998), and 3/ inactivation of eIFiso4G1-2 in Arabidopsis leads to a constitutive heat shock-like response, implying a potential role of eIFiso4G1 as a negative regulator of the heat stress response (Lellis et al. 2010). While these points are intriguing, we want to maintain the virus-centered focus of our manuscript, so we would refrain from adding them in the discussion. We are currently writing a review on the topic, in which we plan to discuss these elements.

Finally, while reviewing the manuscript, we made some additional adjustments, which appear in the text document with tracked changes.

figure legends section has been modified due to figure suppressions/modifications.

Supporting information for all figures are provided as folder in sup data.

**References for Referees answers**

Bi C, Ma Y, Jiang SC, Mei C, Wang XF, Zhang DP. Arabidopsis translation initiation factors eIFiso4G1/2 link repression of mRNA cap-binding complex eIFiso4F assembly with RNA-binding protein SOAR1-mediated ABA signaling. New Phytol. 2019;223: 1388–1406. doi:10.1111/nph.15880

Cho HY, Lu MYJ, Shih MC. The SnRK1-eIFiso4G1 signaling relay regulates the translation of specific mRNAs in Arabidopsis under submergence. New Phytol. 2019;222: 366–381. doi:10.1111/nph.15589

Coutinho de Oliveira, Luciana et al. 2019. “Structural Studies of the EIF4E–VPg Complex Reveal a Direct Competition for Capped RNA: Implications for Translation.” *Proceedings of the National Academy of Sciences*: 201904752. http://www.pnas.org/lookup/doi/10.1073/pnas.1904752116.

Gallie, Daniel R., Hanh Le, Robert L. Tanguay, and Karen S. Browning. 1998. “Translation Initiation Factors Are Differentially Regulated in Cereals during Development and Following Heat Shock.” *Plant Journal* 14(6): 715–22.

Khan, Mateen A. et al. 2006. “Interaction of Genome-Linked Protein (VPg) of Turnip Mosaic Virus with Wheat Germ Translation Initiation Factors EIFiso4E and EIFiso4F.” *Journal of Biological Chemistry* 281(38): 28002–10.

Khan, Mateen A., Hiroshi Miyoshi, Daniel R. Gallie, and Dixie J. Goss. 2008. “Potyvirus Genome-Linked Protein, VPg, Directly Affects Wheat Germ in Vitro Translation: Interactions with Translation Initiation Factors EIF4F and EIFiso4F.” *Journal of Biological Chemistry* 283(3): 1340–49.

Lellis, Andrew D. et al. 2010. “Deletion of the EIFiso4G Subunit of the Arabidopsis EIFiso4F Translation Initiation Complex Impairs Health and Viability.” *Plant Molecular Biology* 74(3): 249–63.

Miyoshi, Hiroshi et al. 2008. “Turnip Mosaic Virus VPg Interacts with Arabidopsis Thaliana EIF(Iso)4E and Inhibits in Vitro Translation.” *Biochimie* 90(10): 1427–34.

Wu HL, Jen J, Hsu PY. What, where, and how: Regulation of translation and the translational landscape in plants. Plant Cell. 2023 Jul 12:koad197. doi: 10.1093/plcell/koad197. Epub ahead of print. PMID: 37437121

Zafirov, Delyan, Nathalie Giovinazzo, Anna Bastet, and Jean Luc Gallois. 2021. “When a Knockout Is an Achilles’ Heel: Resistance to One Potyvirus Species Triggers Hypersusceptibility to Another One in Arabidopsis Thaliana.” *Molecular Plant Pathology* 22(3): 334–47.

.
